# miR-10c Facilitates White Spot Syndrome Virus Infection by Targeting Toll3 in *Litopenaeus vannemei*

**DOI:** 10.3389/fimmu.2021.733730

**Published:** 2021-12-07

**Authors:** Hongliang Zuo, Xinxin Liu, Mengting Luo, Linwei Yang, Zhiming Zhu, Shaoping Weng, Jianguo He, Xiaopeng Xu

**Affiliations:** ^1^ State Key Laboratory of Biocontrol, School of Life Sciences, Sun Yat-Sen University, Guangzhou, China; ^2^ Southern Marine Science and Engineering Guangdong Laboratory (Zhuhai), Zhuhai, China; ^3^ Institute of Aquatic Economic Animals and Guangdong Province Key Laboratory for Aquatic Economic Animals, Sun Yat-Sen University, Guangzhou, China

**Keywords:** microRNA, Toll-like receptor, regulatory factor, antiviral immunity, *Litopenaeus vannemei*

## Abstract

Toll-like receptors (TLRs) are canonical cell membrane receptors functioning to recognize pathogens and transduce signals to activate immune responses. It has been known that Toll3 in Pacific white shrimp *Litopenaeus vannamei* (LvToll3) plays a critical role in antiviral immunity by inducing the transcription of interferon regulatory factor (IRF), which mediates a signaling axis that is similar to the interferon system of vertebrates. However, the regulatory mechanism of the Toll3-IRF signaling is still unclear. In this study, a novel microRNA (miRNA) of miR-10 family, temporarily named as miR-10c, was identified from *L. vannamei*. miR-10c may play a nonnegligible regulatory role in shrimp immune responses since it was constitutively expressed in all detected tissues and transcriptionally induced by immune stimulation. Functional analysis validated that miR-10c could target LvToll3 to inhibit its expression, through which miR-10c blocked the nuclear translocation of IRF and facilitated white spot syndrome virus (WSSV) infection. To our knowledge, the present study revealed the first report of a Toll targeted by miRNA in crustaceans and provided a solid evidence base for supporting the role of LvToll3 in antiviral defense by activating IRF signaling in *L. vannamei*. Identification of the miR-10c/Toll3/IRF regulatory axis in shrimp provides new insights into the participation of miRNA in the regulation of immune responses and contributes to in-depth understanding of the mechanisms of Toll-induced immune responses in *L. vannamei*.

## Introduction

Tolls/Toll-like receptors (TLRs) are one of the most common pathogen sensors in metazoans, recognizing diverse pathogens such as Gram-positive and Gram-negative bacteria, RNA or DNA viruses, fungi, and other protozoans in the early stage of host immune response (1). After pathogen recognition, Tolls transduce signals into cells through various signal pathways to activate expression of a series of immune and inflammatory genes for the establishment of the immune activation state in the host (2). To date, there are 10 and 12 TLRs identified in humans and mice, respectively, in which TLR1, TLR2, TLR5, TLR6, and TLR10 are responsible for microbial lipid, polysaccharide, and protein recognition, while TLR3, TLR7, TLR8, and TLR9 are responsible for viral nucleic acid recognition (3, 4). In invertebrates, the number of Tolls/TLRs varies among different organisms. For example, *Drosophila melanogaster* has nine TLRs, while *Caenorhabditis elegans* has only one and *Paracentrotus lividus* has up to 222 TLRs in their genomes (5–7). Stimulation of TLRs results in the activation of different intracellular signaling cascades, generally leading to the activation of NF-κB and activating protein-1 (AP-1) in MyD88-dependent pathways and type I interferons (IFNs) in TRIF-dependent antiviral pathways (8, 9).

*Litopenaeus vannamei* is the major aquaculture shrimp in the world, the culture of which was threatened by various pathogens, such as *Vibrio*, white spot syndrome virus (WSSV), and *Enterocytozoon hepatopenaei* (EHP), which have caused tremendous economic losses (9, 10). More and more research attentions have focused on the regulation of the innate immune system of *L. vannamei*, which is also centered on the Toll receptor-mediated signaling. Among the identified *L. vannamei* Tolls, the Toll3, highly homologous to *Drosophila* Toll6, has been known to be transcriptionally induced by double-stranded RNA (dsRNA) and can facilitate the expression of interferon regulatory factor (IRF) and the downstream Vago 4/5 (11, 12). It has been known that the IRF-Vago-JAK/STAT regulatory axis plays an essential role in antiviral immunity in shrimp (13). These indicated that LvToll3 may also play a role in virus infection, which still lacks solid evidence so far.

MicroRNAs (miRNAs) are a sort of evolutionary conserved, small (18–26 nt), endogenous noncoding RNAs which transcribed from genomic clusters and involved in multiple biological processes by suppressing the target gene on posttranscriptional level (14). MiRNAs are initially transcribed as primary miRNAs (pri-miRNAs) by RNA polymerase II or III, processed into precursor miRNAs (pre-miRNA) by Drosha/DGCR8 complex in the nucleus, exported to the cytoplasm, and finally cleaved to mature miRNA by Direr complex (15, 16). After incorporating into a RNA-induced silencer complex (RISC), the mature miRNAs combine with target mRNAs and induce their cleavage through the imperfect complementary binding sites located in the 3′-untranslated region (3′-UTR) or open reading frame (ORF) (17, 18). Accumulating evidence proves that miRNAs play nonnegligible regulatory roles in innate immunity by adjusting enzyme activities, regulating apoptosis or phagocytosis, and modulating signal transduction in mammals (19, 20). Recent studies have shown that the miRNA system also plays an important role in regulation of shrimp immunity. For instance, the *L. vannamei* miR-1959 mediates an intrapathway regulatory feedback loop to positively enhance the activation of the dorsal pathway by targeting cactus (21). The miR-1 mediated the inhibitory signaling from the JAK-STAT pathway to the NF-κB signaling by targeting MyD88, the vital signal transducer of dorsal activation (22). In the current study, we identified a novel miRNA from miR-10 family in *L. vannemei* and unveiled its regulatory role in shrimp antiviral immunity *via* targeting LvToll3 and further regulating IRF expression. This study may enrich the knowledge on Toll-mediated signaling in crustaceans and provide new insights into the regulatory effects of miRNAs on immunity in invertebrates (23).

## Materials and Methods

### Shrimps

*L. vannamei* (~5 g) were obtained from an aquaculture farm in Zhuhai, China, acclimated at ~28°C in a recirculating water system filled with air-pumped seawater (5% salinity) and fed with 3% body weight artificial diet for two times each day. Five percent of reared shrimps were sampled randomly and detected by PCR to ensure free of WSSV and *Vibrio parahaemolyticus*.

### Cloning of Mature miR-10c

miR-10c sequence was obtained from a small RNA transcriptome sequencing library and verified using stem-loop real-time RT-PCR following methods as previously reported (21). Briefly, total RNA was extracted from mixed tissues including gill, hepatopancreas, stomach, and muscle of *L. vannamei* using Trizol reagent (Ambion, Austin, TX, USA), and cDNA was then synthesized with stem-loop primer of miR-10c-RT (**Table 1**) using PrimeScript RT reagent kit (Takara, Japan). To verify the sequence of miR-10c, the mature miRNA sequence was cloned and sequenced with primers of miR-10c-IF and miR-10c-IR as previously described (24).

**Table 1:** Primers and probes used in this study.

Name	Sequence (5′–3′)
**miRNA identification**	
miR-10c-RT	GTCGTATCCAGTGCAGGGTCCGAGGTATTCGCACTGGATACGACACACAAG
miR-10c-IF	CTCCAGCTGACCTTGTAGAT
miR-10c-IR	ACCCTGCACTGGATACGAC
**Northern blot and *in situ* hybridization**	
miR-10c probe	/5DigN/ACACAAGTTCGGATCTACAAGGT/3Dig_N/
U6 probe	/5DigN/CACGAUUUUGCGUGUCAUCCUU/3Dig_N/
Scrambled miRNA probe	/5DigN/GUGUAACACGUCUAUACGCCCA/3Dig_N/
**Dual-luciferase reporter assays**	
Toll3-3′-AscIF	AATGGCGCGCCAGATCCTGCGGAACTCCCTG
Toll3-3′-FseIR	ATAGGCCGGCCAAACGACGTGACAATGGTTACAC
Toll3-3′-MutF	ACAGAGCGGAACATTTTGATGTCGGCACAAGCATAATCTCAGAGCTCTACCTAG
Toll3-3′-MutR	TTGTGCCGACATCAAAATGTTCCGCTCTGTGATGAAAGCTCCTGGCTGTG
**dsRNA synthesis**	
Toll3-dsT7F	GGATCCTAATACGACTCACTATAGGGCCTGTGATTGCGAGATGAC
Toll3-dsT7R	GGATCCTAATACGACTCACTATAGGAGGATAACCACGACGACGAAG
Toll3-dsF	GCCTGTGATTGCGAGATGAC
Toll3-dsR	AGGATAACCACGACGACGAAG
GFP-dsT7F	GGATCCTAATACGACTCACTATAGGATGGTGAGCAAGGGCGAGGAG
GFP-dsT7R	GGATCCTAATACGACTCACTATAGGTTACTTGTACAGCTCGTCCATGCC
GFP-dsF	ATGGTGAGCAAGGGCGAGGAG
GFP-dsR	TTACTTGTACAGCTCGTCCATGCC
**qRT-PCR**	
U6-RT	AAATGTGGAACGCTTCAC
U6-qRTF	GTACTTGCTTCGGCAGTACATATAC
U6-qRTR	TGGAACGCTTCACGATTTTGC
10c-qRTF	CTCCAGCTGACCTTGTAGATC
10c-qRTR	ACCCTGCACTGGATACGAC
Toll3-qRTF	TTCAGAACAGCCAGCGAGTG
Toll3-qRTR	GCATTGACGCTGGACTGTTG
IRF-qRTF	ATCCAACCTGTCTTCAGTGGAG
IRF-qRTR	GGACCACGCTGTGAACCTG
Vago4-qRTF	GAAGTGCTGGCTGCCCAAG
Vago4-qRTR	GACCGCATGTAGCATACTCGAC
Vago5-qRTF	CTCTCCAACATCTGATCGCAG
Vago5-qRTR	CAGTGTGCCCGTACACAGC
ALF2-qRTF	TAGCGTGACACCGAAATTCAAG
ALF2-qRTR	CGAAGTCTTGCGTAGTTCTGC
ALF3-qRTF	CGGTGACATTGACCTCGTTG
ALF3-qRTR	TGACGGACCCGATGAAGTAG
ALF5-qRTF	TGGTGAAGGCTTCCTACAAGAG
ALF5-qRTR	CATCAGCAGTAGCAGTGTCA
ie1-qRTF	GCCATGAAATGGATGGCTAGG
ie1-qRTR	ACCTTTGCACCAATTGCTAGTAG

### Quantitative Real-Time PCR

For expression analysis of miRNA, total RNA of different samples was extracted using Trizol reagent (Ambion), and cDNAs were synthesized using stem-loop primer of miR-10c-RT as mentioned above. Small nuclear RNA U6 was separately reversed using primers of U6-RT (**Table 1**) and set as internal control. For expression analysis of mRNA, total RNA of different samples were extracted using a RNeasy Plus Mini Kit (QIAGEN, Los Angeles, CA, USA), cDNAs were synthesized using a PrimeScript RRT Kit (Takara, Japan), and shrimp elongation factor 1-alpha (EF1α, GenBank Accession No. GU136229.1) gene was set as internal control. Quantitative real-time PCR (qRT-PCR) was performed on a LightCycler 480 System (Roche, Basel, Switzerland) using specific primers list in **Table 1**, and the qRT-PCR protocols and parameters were set as previously described (21). The expression levels of miRNA and mRNA were calculated using 2^−ΔΔCt^ method after normalization to U6 and EF1α, respectively (25).

### Northern Blot

After pre-electrophoresis of a 12% denaturing polyacrylamide gel at 200 V for 1 h, total RNAs (10 μg) isolated from different samples were separated at 200 V for 1 h and transferred onto a positively charged nylon membrane (Roche, USA) using a Trans-Blot SD Semi-Dry Transfer Cell system (Bio-Rad, Hercules, CA, USA). Separated RNAs were crosslinked to the membrane by UV (254 nm) and prehybridized at 68°C for 30 min using ULTRAhyb Ultrasensitive Hybridization Buffer (Ambion, USA), and hybridized overnight with miR-10c and U6 LNA probes (Exqion, Copenhagen, Denmark, **Table 1**) at a final concentration of 0.1 nM at 60°C, respectively. Membranes were blocked by DIG Wash and Block Buffer Set (Roche, USA) and incubated with 1:10,000 diluted Anti-Digoxigenin-AP, Fab fragments (Roche, USA). The signals were detected using CDP-Star (Roche, USA) chemiluminescent substrate and captured by Amersham Imager 600 (GE, Chicago, IL, USA).

### *In Situ* Hybridization

The gill and hepatopancreas of healthy shrimps were fixed in 4% paraformaldehyde, dissolved by PBS (0.1 M, pH: 7.4) overnight and embedded in paraffin. The embedded tissues were cut into 5 μm thick paraffin slices and fixed onto silicified slides. *In situ* hybridization was performed using miRCURY LNA miRNA ISH optimization kit (Exqion, Denmark) according to the procedures mentioned previously (21). In brief, slides were deparaffinized in xylene and gradient-diluted ethanol solutions at room temperature (RT). The preprocessed slides were then incubated with proteinase-K (10 mg/ml) for 15 min at 37°C, washed with PBS, dehydrated in gradient-diluted ethanol solutions, and air dried for 15 min at RT. Dehydrated tissues were incubated with hybridization buffer containing 20 nM miR-10c probes, 5 nM U6 probes, or 20 nM scrambled miRNA probes (Exiqon, Denmark) for 1 h at 50°C in a humidifying chamber. Slides were then washed in SSC buffers at the hybridization temperature, blocked with a blocking solution for 15 min at RT, incubated with 1:500 diluted Anti-Digoxigenin-AP, Fab fragments (Roche, USA) for 1 h at RT, developed with NBT/BCIP (Roche, USA) substrate for 2 h at 30°C, counterstained with Nuclear Fast Red (Sigma, St. Louis, MO, USA) for 1 min at RT, and finally captured using a Leica DM4 light microscopy (Leica, Wetzlar, Germany).

### Immune Challenge

After culturing to logarithmic phase, *V. parahaemolticus* was diluted to 10^5^ colony forming unit (CFU) in 50 μl PBS. WSSV used in this study was prepared from moribund shrimps artificially infected with the preserved WSSV that was stored in −80°C in our lab. Virus stock was prepared freshly and quantified using absolute qRT-PCR and diluted to 10^6^ copies in 50 μl PBS according to procedure as mentioned previously (26). Five micrograms of litopolysaccharide (LPS) or poly(I:C) was diluted in 50 μl PBS before use as the experimental inoculum.

Healthy shrimps were divided into several experimental groups and acclimated in independent recirculating water tank systems for 1 week before immune challenge. For expression pattern analysis of miR-10c after pathogen stimulation, shrimps were injected with 10^5^ CFU of *V. parahaemolticus*, 10^6^ copies of WSSV fresh extracted WSSV, 5 μg LPS, 5 μg poly(I:C), and PBS at the second abdominal segment. The gill and hepatopancreas were isolated from six randomly sampled shrimps in each group at 0, 4, 12, 24, 48, 72, and 96 h poststimulant injection. Total RNAs were then isolated, cDNAs were synthesized by stem loop primer, and expression of miR-10c was detected using qRT-PCR as described above.

### Dual-Luciferase Reporter Assays

The wild-type 3′-UTR of LvToll3 was cloned using primers of Toll3-3′-AscIF/Toll3-3′-AscIR (**Table 1**) and inserted into pGLDr249, a dual-luciferase miRNA target expression vector constructed from pmirGLO vector (Promega, USA), after the ORF of luciferase to generate pGLDr249-Toll3 vector. Since both 5′ and 3′ end of miRNAs potentially dominant the target sites, the whole predicted target site of miR-10c in LvToll3 (5’ ACACGGCUGUAGUUUUACAAGG 3′) was mutated to complement sequence (5′ UGUGCCGACAUCAAAAAUGUUCC 3′) using primers of Toll3-3′-MutF/Toll3-3′-MutR (**Table 1**) and constructed into pGLDr-249 to generate pGLDr249-Toll3-Mut.

To verify the posttranscriptional inhibiting effects of miR-10c *via* the target site in 3′-UTR of LvToll3, *Drosophilla* (S2) cells were plated on a 96-well plate with 80% confluent and acclimated overnight at 28°C in Schneider’s insect medium (Sigma, USA) in the presence of 10% serum (Gibco, Waltham, MA, USA). For each well, 100 ng pGLDr249-Toll3 or pGLDr249-Toll3-Mut was cotransfected with 15 pmol miR-10c mimics or miR-NC (GenePharma, Shanghai, China) (final concentration of 100 nM) into S2 cells using FuGENE^®^ HD Transfection Regent (Promega, USA). Cells were harvested and lysed at 48 h posttransfection, and the activity of luciferase was detected using Dual-Luciferase^®^ Reporter Assay System (Promega, USA) according to the instruction of manufactures. Parallel with the luminescence detection, the protein levels of firefly and Renilla luciferases in the cell lysates were verified by Western blot using specific antibodies against firefly and Renilla luciferases (Abcam, USA), respectively.

### miRNA Regulation and mRNA Knockdown *In Vivo*

To verify the posttranscriptional suppression of LvToll3 *in vivo*, *L. vannamei* were treated with chemosynthetic and cholesterol-modified miR-10c mimics (agomiR-10c) or miR-10c inhibitor (antagomiR-10c). Healthy shrimps were randomly divided into four groups (*n* = 40 in each group) and injected with agomiR-10c, agomiR-NC, antagomiR-10c, or antagomiR-NC at the second abdominal. Hemocytes and gills were pooled from 15 shrimps of each treatment at 48 h postinjection for the following transcriptional and proteomic analyses.

The expression of LvToll3 was inhibited by RNA interference *via* dsRNA injection *in vivo*. The dsRNAs targeting LvToll3 or green fluorescent protein (GFP, set as negative control) were synthesized *in vitro* using T7 RiboMAX™ Express RNAi system (Promega, USA). The template sequence of LvToll3 and GFP were cloned from the cDNA of *L. vannamei* and pAc5.1-GFP plasmid and incorporated with T7 RNA polymerase promoter at the 5′ end using primers of Toll3-dsF/Toll3-dsR and GFP-dsF/GFP-dsR (**Table 1**), respectively. The target-specific dsRNAs were annealed from two independently transcribed single-strand RNAs and then purified according to the manufacturer’s instruction. Two groups (*n* = 40) of healthy shrimps were intramuscularly injected with 5 μg LvToll3 and GFP dsRNA, respectively. At 48 h post-dsRNA injection, hemocytes and gills were sampled from 15 shrimps in each group for the following mRNA and protein analyses as described above.

### Nuclear Localization Analysis of IRF

At 48 h post-miRNA or dsRNA injection, shrimps were challenged with poly(I:C) as mentioned above and 24 h later, samples were pooled and subjected to Western blot analysis. In brief, total nuclear protein of hemocyte and gill, dispersed through 200 mesh screen, was isolated using Nuclear and Cytoplasmic Protein Extraction Kit (Beyotime, Haimen, China), and then quantified and diluted to equal concentration using BCA protein Assay kit (Beyotime, China). Protein samples were separated by SDS-PAGE and transferred onto a nitrocellulose membrane (GE, USA). After blocking, membrane was incubated with prepared rabbit anti-LvToll3 Ab (GL Biochem, Shanghai, China), and simultaneously, a parallel membrane was incubated with rabbit anti-HistoneH3 mAb (CST, Houston, TX, USA) as internal control. Another parallel membrane was incubated with rabbit anti-GAPDH antibody (Sigma, USA) to verify no contamination of cytoplasmic protein. The signals were detected using anti-rabbit IgG (H+L)-HRP Conjugate (Promega, USA), developed using SuperSignal West Femto (Thermo, USA) and captured by Amersham Imager 600 (GE, USA). The gray values of visualized Western blot signals were quantitated using Quantity one (Bio-Rad, USA) by Gauss model with different rolling disk sizes (5, 10, and 15).

### Analysis of the Antiviral Function of miR-10c and LvToll3

To analyze the immune status of miR-10c, shrimps were injected with miRNA mimics and inhibitor as mentioned above. Gills were sampled from nine shrimps in each group at 48 h after injection. Total RNAs were isolated, cDNAs were synthesized, and then the transcription of immune-related genes, including Vagos, anti-lipopolysaccharide factors (ALF), and C-type lectins (CTL), were analyzed using qRT-PCR with specific primers (**Table 1**). The *L. vannamei* elongation factor 1-α (EF1-α) (GenBank Accession No.: GU136229) was set as internal control.

Shrimps (*n* = 50) were challenged with 10^6^ copies of WSSV at 48 h after the injection of miR-10c mimics/inhibitor or LvToll3 dsRNA as mentioned above. The cumulative mortality was recorded days after WSSV challenge. Parallel experiments were performed to detect the WSSV copy number in muscle after the immune challenge. To verify the role of antiviral immunity of miR-10c, shrimps were coinjected with miRNA mimics and dsRNA and challenged with 10^6^ copies of WSSV at 48 h postinjection. The cumulative mortality was recorded and parallel experiments were performed to detect the WSSV copy number in muscle. Total RNA of muscles was isolated and the WSSV copies were detected by relative qRT-PCR with the EF1-α as internal control.

### Bioinformatics Analysis and Statistical Analysis

The target of miR-10c was predicted using RNAhybrid (https://bibiserv.cebitec.uni-bielefeld.de/rnahybrid/submission.html) based on the mRNA transcriptome of *L. vannamei* with the parameter of hits per target of 3, energy threshold of −25, No G:C in seed, helix constraint from 2 to 8, max bulge loop length of 3, mas internal loop length of 3, and approximate *p*-value of 3utr_fly (27). The multiple sequence alignment of miR-10 homologs was performed using ClustalX 2.1, and the phylogenetic tree was constructed using MEGA 5.0. All data were presented as mean ± SD. The significance of difference between groups of numerical data was calculated using Student’s *t*-test. The cumulative mortalities were analyzed using GraphPad Prism 5.01 to generate the Kaplan-Meier plot (log rank *χ* ^2^ test).

## Results

### miR-10c Identification

**Figure 1:**
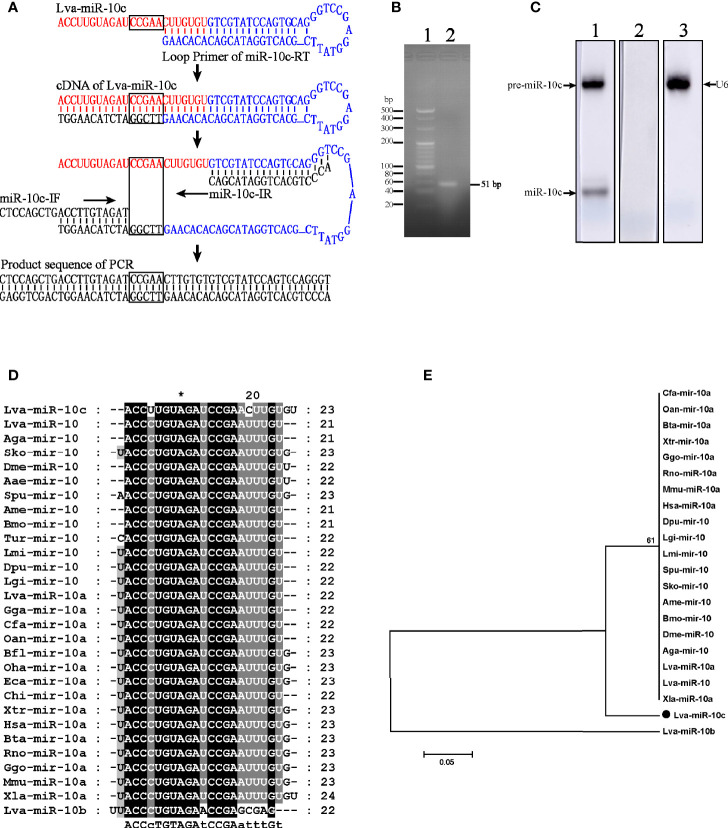
Identification and homology analysis of Lva-miR-10c. **(A)** Schematic diagram of the verification of miR-10c by stem-loop RT-PCR. The mature miR-10c and stem-loop primer were marked in red and blue, respectively. The base pairs for identification were framed. **(B)** Electrophoresis of the stem-loop RT-PCR product. Lane 1, 20 bp DNA ladder; lane 2, the PCR product. **(C)** Northern blot analysis of miR-10c in total RNA of *L. vannamei*. Lane 1, miR-10c-specific probe; lane 2, scrambled probe; lane 3, U6-specific probe. **(D)** Multiple sequence alignment of miR-10 homologs. **(E)** Phylogenetic tree of miR-10 homologs. Sequences analyzed include *L. vannamei* miRNAs from GenBank: Lva-miR-10c (GenBank Accession No. MZ462071), Lva-miR-10a (MZ462069), and miR-10b (MZ462070) and other miRNAs from miRbase: Lva-miR-10, *L. vannamei* (miRbase Accession No. MIMAT0032193); Aga-miR-10, *Anopheles gambiae* (MIMAT0001497); Sko-mir-10, *Saccoglossus kowalevskii* (MIMAT0004419); Lgi-mir-10, *Lottia gigantea* (MIMAT0009563); Dpu-mir-10, *Daphnia pulex* (MIMAT0012636); Lmi-mir-10, *Locusta migratoria* (MIMAT0010144); Aga-mir-10, *Anopheles gambiae* (MIMAT0001497); Spu-mir-10 *Strongylocentrotus purpuratus* (MIMAT0009654); Bmo-mir-10, *Bombyx mori* (MIMAT0004195); Dme-miR-10, *Drosophila melanogaster* (MIMAT0000115); Oan-mir-10a, *Ornithorhynchus anatinus* (MIMAT0007122); Gga-mir-10a, *Gallus gallus* (MIMAT0007731); Cfa-mir-10a, *Canis familiaris* (MIMAT0006737); Hsa-miR-10a, *Homo sapiens* (MIMAT0000253); Xtr-mir-10a, *Xenopus tropicalis* (MIMAT0003557); Mmu-miR-10a, *Mus musculus* (MIMAT0000648); Bta-mir-10a, *Bos taurus* (MIMAT0003786); Rno-miR-10a, *Rattus norvegicus* (MIMAT0000782); Ggo-mir-10a, *Gorilla gorilla* (MIMAT0002486); and *Xla-miR-10a, Xenopus laevis* (MIMAT0046422).

To investigate miRNA profiles of *L. vannemei* during the responses to virus, small RNAs were isolated from hemocytes at 4, 12, 24, and 48 h after the intramuscular injection of WSSV and the miRNA libraries were constructed and sequenced on the Illumina HiSeq 2000 sequencing platform. Two hundred eighty-eight differentially expressed miRNAs (DEmiRNAs) had been classified as a data set (**Supplementary File 1**). In this data set, the richness of a small RNA sequence (5′ ACCTTGTAGATCCGAACTTGTGT 3′), highly homologous to the miR-10a of *Xenopus laevis* and temporarily named Lva-miR-10c, was increased from 1,057 to 5,879 reads at 4 to 48 h post WSSV injection. The mature sequence of miR-10c was verified by the five identified base pairs using stem-loop RT-PCR. Generally, the cDNA of miR-10c was reverse transcribed by stem-loop primer of miR-10c-RT through the combination between its unpaired 7 nucleotides and the 3′ end of miR-10c (**Figure 1A**). After then, the stem-loop conjugated miR-10c was amplified by the identification primers of miR-10c-IF and miR-10-IR with partial complementation of the forward primer and complete complementation of the reverse primer. PCR products were purified through agarose gel electrophoresis (**Figure 1B**) and sequenced after cloning in *Escherichia coli* system. The accuracy of the cloning results was verified by the uncombined sequence in miR-10c (black box in **Figure 1A**). Northern blot further confirmed that miR-10c and its precursor were expressed in *L. vannemei* with the control of small nuclear RNA U6 (**Figure 1C**). Homology analysis demonstrated that miR-10c shared high homology with the miR-10/miR-10a family from vertebrates and invertebrates with 2 nt of difference at positions of 4 and 17 (**Figure 1D**), and it was named after Lva-miR-10b following the principles presented in Ambros’s study (28). The phylogenetic tree also revealed that miR-10c was located at the distinct evolutionary branch from the analyzed miR-10, miR-10a, and miR-10b (**Figure 1E**). These results suggested that miR-10c could be a novel identified miRNA from the miR-10 family of *L. vannamei*.

### The Distribution of miR-10c in *L. vannamei*

**Figure 2:**
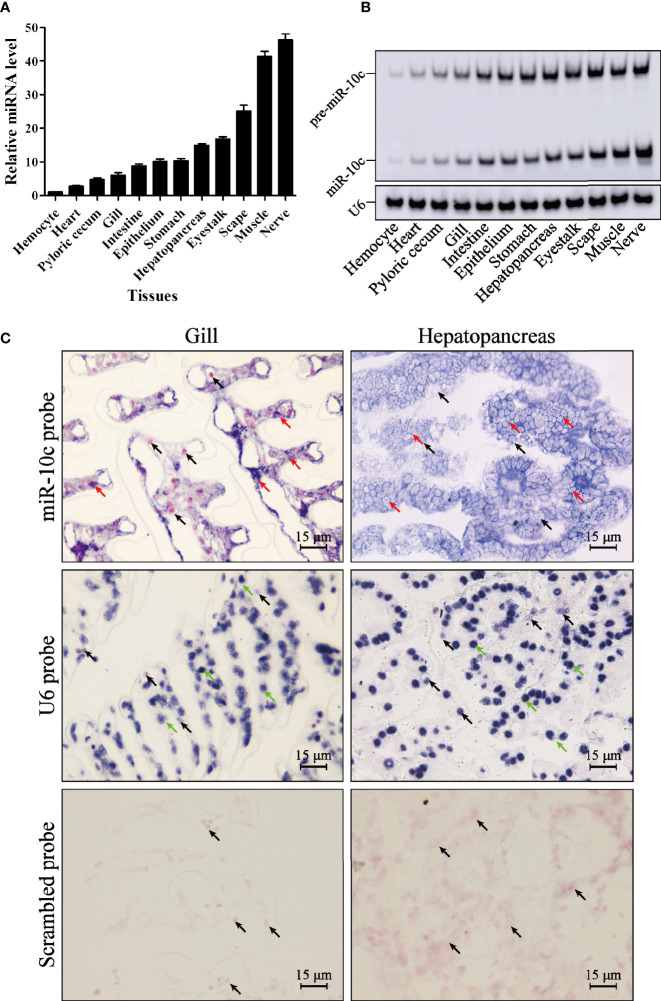
MiR-10c distribution in *L. vannamei* tissues. **(A)** Expression of miR-10c in *L. vannamei* tissues detected by stem-loop qRT-PCR with U6 RNA as internal control. The expression level of miR-10c in hemocyte was set as baseline (1.0). **(B)** The tissue distribution of miR-10c analyzed by Northern blot. **(C)** *In situ* hybridization analysis of miR-10c in hepatopancreas and gill tissues. The signals of miR-10c (red arrows) and U6 RNA (green arrows) were colored dark blue and the nuclei were counterstained red by Nuclear Fast Red (black arrows).

The expression level of miR-10c in 12 tissues of *L. vannamei* was detected by stem-loop qRT-PCR (**Figure 2A**). Results showed that miR-10c could be detected in all examined tissues, with the lowest expression level in hemocyte. The level of miR-10c in heart, pyloric cecum, gill, intestine, epithelium, stomach, hepatopancreas, and eyestalk was 2.75-, 4.71-, 5.98-, 8.77-, 10.13-, 14.94-, and 16.78-fold over that in hemocyte, respectively. MiR-10c was highly expressed in scape, muscle, and nerve, which was 22.63-, 39.95-, and 41.93-fold over that in hemocyte. The results were further verified by Northern blot (**Figure 2B**). Besides, results of *in situ* hybridization showed that the expression of miR-10c, mainly detected in cytoplasm, was visually higher in hepatopancreas than in gill (**Figure 2C**). As a positive control, the U6 RNA was mainly detected in the nucleus.

### Expression Profiles of miR-10c After Immune Stimulation

**Figure 3:**
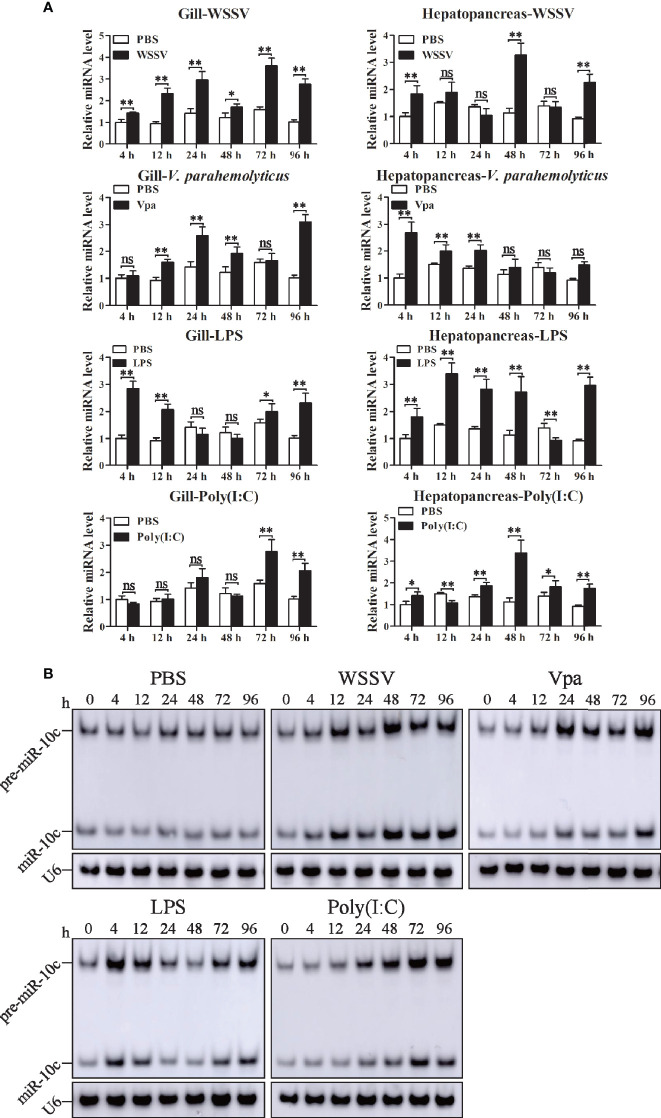
The expression of miR-10c after immune stimulation. **(A)** Stem-loop qRT-PCR analysis of miR-10c expression in gill and hepatopancreas of PBS (negative control), WSSV, *V. parahaemolticus* (Vpa), LPS, and poly(I:C)-stimulated shrimp. The U6 RNA was used as internal control. Data are representative of three experiments and presented as means ± SD of four parallel detections. In each panel, the value at 0 h was set as the baseline (1.0). Each bar represents the mean ± SD (*n* = 4), ^*^ *p* < 0.05, ^**^ *p* < 0.01, and ns > 0.05 by one-way ANOVA with Dunnett’s *post-hoc* test compared with 0 h. **(B)** Northern blot analysis of miR-10c expression after stimulations in gill. The expression level of miR-10c was detected at 0, 4, 12, 24, 48, 72, and 96 h postinjection of PBS, WSSV, *V. parahaemolticus* (Vpa), LPS, and poly(I:C).

The expression profiles of miR-10c in gill and hepatopancreas were detected after the injection of WSSV, *V. parahaemolticus*, LPS, and poly(I:C) (**Figures 3A, B**). The expression of miR-10c was slightly and irregularly changed in both gill and hepatopancreas after PBS injection. Upon WSSV stimulation, compared with the PBS control at the same time points, miR-10c was significantly upregulated to 1.43-, 2.51-, 2.09-, 1.49-, 2.28-, and 2.71-fold at 4, 12, 24, 48, 72, and 96 h postinjection (hpi) in gill, respectively, while it was periodical upregulated in hepatopancreas with the two peaks of 2.88- and 2.46-fold at 48 and 96 hpi, respectively. Similar to WSSV, the viral mimic poly(I:C) significantly upregulated the expression of miR-10c in gill during the later period of stimulation, which reached to the peak of 2.03-fold at 96 hpi, however, it showed no solid activatory effect on miR-10c expression in hepatopancreas except a peak of 2.98-fold at 48 hpi. In response to *V. parahaemolticus* (Vpa) infection, the expression of miR-10c in gill and hepatopancreas were generally upregulated and showed diverse expression patterns. It was activated at the late stage in gill and the early stage in hepatopancreas after infection. After the stimulation with LPS, the major pathogen-associated molecular pattern (PAMP) of bacterial pathogens, expression of miR-10c was oscillatorily activated in gill with two peaks of 2.84- and 2.28-fold at 4 and 96 hpi, respectively, which differed from the prolonged activation in hepatopancreas. Similar expression profiles of miR-10c in gill after immune stimulation were verified by Northern blot, which were generally consistent with the results of stem-loop qRT-PCR as mentioned above (**Figure 3C**).

### Target Identification of miR-10c

**Figure 4:**
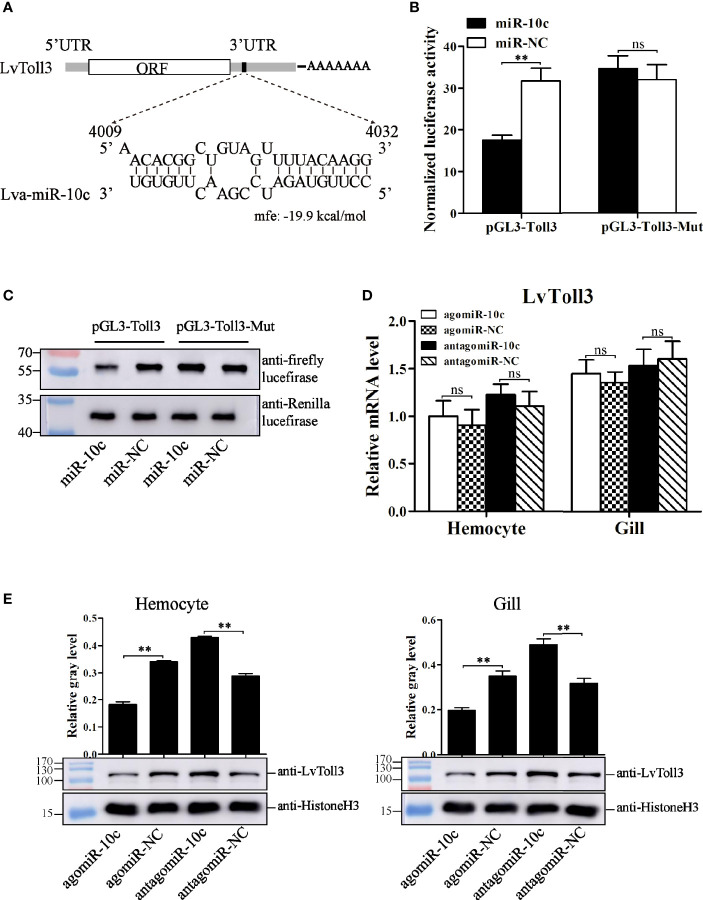
Target identification of miR-10c. **(A)** Scheme of the predicted target site of miR-10c in the 3′-UTR of LvToll3. **(B)** Dual-luciferase reporter assay analysis of the inhibitory effect of miR-10c mimics and its control on the 3′-UTR of LvToll3 and miR-10c binding site-mutated 3′-UTR of LvToll3 (Toll3-Mut). Each bar represents the mean ± SD (*n* = 8), ^**^ *p* < 0.01 and *p > 0.05 by two-tailed unpaired Student’s *t*-test. **(C)** Western blot analysis of the protein level of firefly and Rellina luciferases in the cell lysates of the dual-luciferase reporter assay. **(D)** qRT-PCR analysis of the effects of miR-10c mimics (agomiR-10c) and inhibitor (antagomiR-10c) on mRNA level of LvToll3 in hemocyte and gill. Each bar represents the mean ± SD (*n* = 4), ns > 0.05 by two-tailed unpaired Student’s *t*-test. **(E)** Western blot analysis of the protein level of LvToll3 in hemocyte and gill after treatment with miR-10c mimics and inhibitor in shrimp. The protein level of LvToll3 protein bands were normalized to those of the internal control Histone H3. Each bar is mean ± SD of three independent quantification of the electrophoretic bands, ^**^ *p* < 0.01 by two-tailed unpaired Student’s *t*-test.

To reveal the regulatory role of miR-10c in immunity, 3′-UTR sequence of many reported genes (**Supplementary File 2**) that play regulatory role in the antiviral immunity of *L. vannaemi* was gathered and formatted as a target database to screen the target site of miR-10c. Results demonstrated that miR-10c was incompletely complementary with the 3′-UTR of LvToll3 mRNA (**Figure 4A**). Dual-luciferase reporter assays proved that miR-10c suppressed the expression level of firefly luciferase, the ORF of which was suffixed with the wild-type 3′-UTR of LvToll3, by 44.8% (**Figure 4B**). Western blot also showed that the protein level of firefly luciferase in the dual-luciferase reporter assay was significantly decreased compared with the control (**Figure 4C**). In contrast, both miR-10c and miR-NC mimics showed no effects on the expression of luciferase suffixed with the miR-10c target mutated 3′-UTR of LvToll3 (Toll3-Mut). To validate the suppression of LvToll3 by miR-10c, shrimp were treated with miR-10c mimics or inhibitor and the expression of LvToll3 was analyzed. As the results demonstrated (**Figures 4D, E**), although the mRNA level of LvToll3 showed no obvious change after treatment with miR-10c mimics/inhibitor both in hemocyte and gill, the protein level of LvToll3 was significantly decreased by 46.5% and 44.0% compared with the control mimics (agomiR-NC) in hemocyte, and increased by 48.9% and 54.7% compared with the control inhibitor (antagomiR-NC) in hemocyte and gill, respectively. These results confirmed that miR-10c could target the LvToll3 gene at posttranscriptional level. To further investigate the correlationship between miR-10c and LvToll3, the protein level of LvToll3 in gill was detected using Western blot after immune stimulation (**Supplementary Figure S1**). Results showed that expression of LvToll3 was gradually decreased after the PBS injection, which was generally contrary the expression trend of miR-10c (**Figure 3**). However, the expression of LvToll3 was gradually increased after WSSV injection and showed a similar expression trend with that of miR-10c. The opposite expression trend of miR-10c and LvToll3 was only observed at 96 h after the injection of *V. parahaemolyticus*. These imply that the expression of LvToll3 may be regulated by diverse factors more than miR-10c.

### Regulatory Effects of miR-10c on the Function of IRF

**Figure 5:**
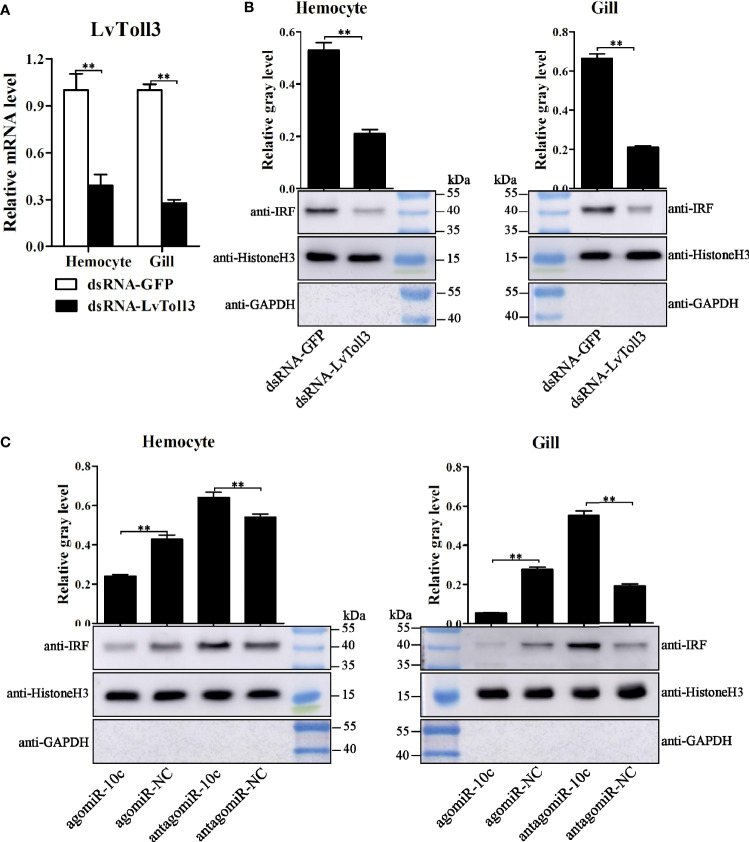
IRF nuclear-translocation regulated by LvToll3 and miR-10c. **(A)** qRT-PCR analysis of the knockdown efficiency of LvToll3. Values in the dsRNA-GFP control group were set as the baseline (1.0). Each bar represents the mean ± SD (*n* = 4), ^**^ *p* < 0.01 by two-tailed unpaired Student’s *t*-test. **(B**, **C)** Western blot analysis of the nuclear translocation of IRF after treatment with miR-10c mimics/inhibitor *in vivo*. The gray values of IRF (41.0 kDa) bands were normalized to those of the nuclear internal control of Histone H3 (15.4 kDa). GAPDH (35.5 kDa) protein was detected to verify no contamination of cytoplasmic protein. Each bar is mean ± SD of three independent quantification of the electrophoretic bands, ^**^ *p* < 0.01 by two-tailed unpaired Student’s *t*-test.

It had been corroborated that LvToll3 could facilitate the transcription of IRF, a transcription factor with a critical role in antiviral immunity of *L. vannamei* (11). In this study, the relationship between LvToll3 and IRF was further investigated *in vivo*. Shrimps were injected with dsRNA-GFP and dsRNA-Toll3, and 48 h later were further stimulated with poly(I:C). Compared with that in the control group, expression of LvToll3 was significantly downregulated by 61.9% and 73.1% in hemocyte and gill, respectively (**Figure 5A**). After knockdown of LvToll3 and stimulation of poly(I:C), total nuclear protein was isolated from hemocytes and gills and the protein level of IRF was analyzed by Western blot. Results demonstrated that the level of nuclear-translocated IRF, normalized to the nuclear internal control of Histone H3, was significantly decreased by 60.2% and 68.2% in hemocyte and gill compared with the control, respectively (**Figure 5B**). Consistently, the nuclear-translocated IRF was decreased by 44.0% and 80.0% after the treatment of miR-10c mimics in hemocyte and gill compared with the control, respectively (**Figure 5C**). By contrast, the nuclear-translocated IRF was increased by 18.5% and 189.7% after the inhibition of miR-10c in hemocyte and gill, respectively. These results elucidate that miR-10c may attenuate the transcriptional regulatory function of IRF by targeting LvToll3.

The NF-κB pathways are important signaling channels downstream of many Tolls/TLRs in animals. We further explored the regulatory relationship between miR-10c and dorsal, a NF-κB family member in shrimp (**Supplementary Figure S2**). After dorsal silencing in shrimp (**Supplementary Figure 2A**), expression of miR-10c did not change significantly (**Supplementary Figure 2B**). The miR-10c mimics and inhibitors could not affect expression of Dorsal *in vivo* as well (**Supplementary Figure 3C**). These were consistent with a previous study that Dorsal could not be a downstream transcription factor of LvToll3 in shrimp (12).

### Roles of miR-10c and LvToll3 in Antiviral Immunity

**Figure 6:**
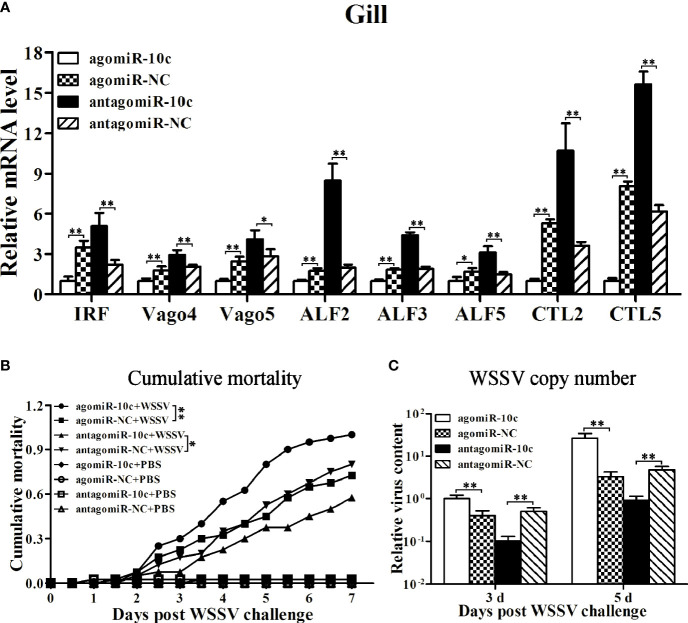
The roles of miR-10c in antiviral immunity. **(A)** qRT-PCR analysis of effects on mRNA level of immune-related genes in gill regulated by miR-10c. Values in the agomiR-10c group were set as the baseline (1.0). Each bar represents the mean ± SD (*n* = 4), ^**^ *p* < 0.01 and ^*^ *p* < 0.05 by two-tailed unpaired Student’s *t*-test. **(B)** Cumulative mortality of miR-10c mimics/inhibitor-treated shrimps after WSSV infection. Data were recorded every 4 h and ^**^ *p* < 0.01 and ^*^ *p* < 0.05 by Kaplan-Meier log-rank *χ* ^2^ tests. **(C)** Relative viral copy numbers in muscle analyzed by qRT-PCR with the DNA of *EF1-α* gene as the internal control. The value in agomiR-10c-treated group at 3 days was set as baseline (1.0). Each bar represents the mean ± SD (*n* = 4), ^**^ *p* < 0.01 by two-tailed unpaired Student’s *t*-test.

The above results indicated a regulatory cascade of miR-10c/Toll3/IRF in shrimp. The role of miR-10c and LvToll3 in antiviral immunity was further investigated *in vivo*. The expression of IRF, vago 4/5, and a set of canonical immune effector genes in gill were detected by qRT-PCR. Results demonstrated that the expression of IRF, Vago4/5, ALF2, ALF3, ALF5, CTL2, and CTL5 was decreased by treatment with miR-10c mimics and increased by the inhibition of miR-10c (**Figure 6A**). Notably, it had been validated that the transcription of Vago4/5 can be activated by IRF in *L. vannamei* (13). The effects of miR-10c on the transcription of IRF and Vago4/5 may indicate that miR-10c could suppress the regulatory cascade mediated by IRF.

**Figure 7:**
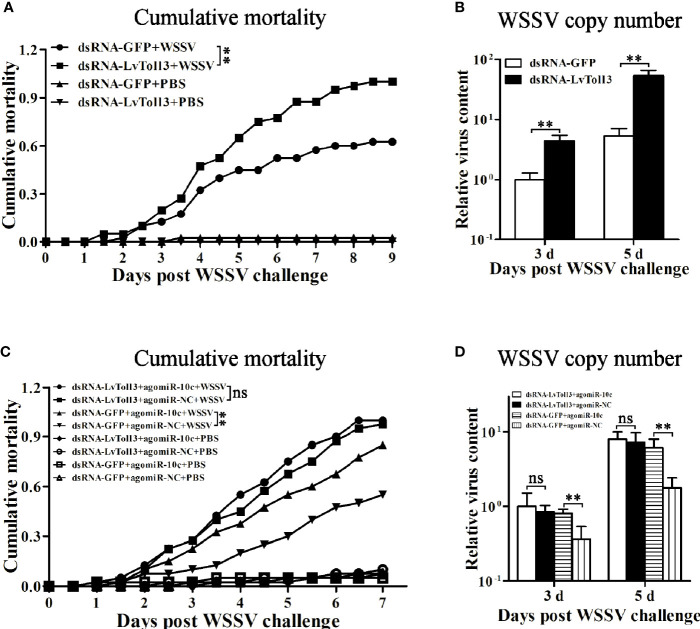
Validation of LvToll3-mediated antiviral immunity regulated by miR-10c. **(A)** Cumulative mortality of LvToll3-knockdown shrimps after WSSV infection. Data were recorded every 4 h and ^**^ *p* < 0.01 by Kaplan-Meier log-rank *χ* ^2^ tests. **(B)** Relative viral copy numbers in muscle were analyzed by qRT-PCR with the DNA of *EF1-α* gene as the internal control. The value in agomiR-10c treated group at 3 days was set as baseline (1.0). Each bar represents the mean ± SD (*n* = 4), ^**^ *p* < 0.01 by two-tailed unpaired Student’s *t*-test. **(C)** Cumulative mortality of dsRNA- and miRNA mimic-coinjected shrimps after WSSV infection. Data were recorded every 4 h and ^**^ *p* < 0.01 and ns > 0.05 by Kaplan-Meier log-rank *χ* ^2^ tests. **(D)** Relative viral copy numbers in muscle analyzed by qRT-PCR with the DNA of *EF1-α* gene as the internal control. The value in LvToll3 dsRNA and miR-10c mimic-coinjected group at 3 days was set as baseline (1.0). Each bar represents the mean ± SD (*n* = 4), ^**^ *p* < 0.01 by two-tailed unpaired Student’s *t*-test.

The role of miR-10c in antiviral immunity was further investigated (**Figures 6B, C**). Comparing with the control mimics, miR-10c mimics significantly increased the cumulative mortality of the WSSV-infected shrimp by 37.9% at 7 days postinfection (dpi) and elevated the virus loads in muscle by 148.4% and 698.6% at 3 and 5 dpi, respectively. On the contrary, comparing with the control inhibitor, miR-10c inhibitor alleviated the virulence of WSSV and decreased the cumulative mortality by 28.1% at 7 dpi and reduced the virus loads in muscle by 79.8% and 80.8% at 3 and 5 dpi, respectively. Consistent with these, after the knockdown of LvToll3, the cumulative mortality was significantly increased by 60% at 9 dpi and the virus loads in muscle were elevated by 339.2% and 914.4% at 3 and 5 dpi compared with GFP control, respectively(**Figures 7A, B**).

### miR-10c Regulates the Antiviral Immunity of Shrimp Through Targeting LvToll3

To investigate whether the attenuation of shrimp antiviral immunity by LvmiR-10c is mediated by LvToll3 (**Figures 7C, D**), dsRNA and miRNA mimics were coinjected into shrimp, which were further challenged with WSSV. Results demonstrated that in the control GFP dsRNA-treated group, the cumulative mortality was increased by 54.5% after miR-10c mimic treatment compared with the mimic control. Accordingly, the virus load in muscle was increased by 119.2% and 242.8% at 3 and 5 dpi in the miR-10c mimic treatment group, respectively. In contrast, in the LvToll3-silenced group, there was no significant change of the cumulative mortality of shrimp after miR-10c mimic treatment compared with the mimic control. Although the virus load in the Toll3-dsRNA/miR-10c mimics together-treated shrimp was slightly increased by 18.5% and 10.2% at 3 and 5 dpi compared with the control, respectively, there was no statistically difference between the control and experimental groups. These suggested that LvToll3 is essential for the role of LvmiR-10c in antiviral immunity.

## Discussion

At present, more than 23,365 known miRNAs and 481 novel miRNA candidates have been reported in *L. vannamei* in total, several of which are known or predicted to be involved in virus infection, cold or heat adaption, ER stress, and hypoxia stimulation (29–31). Three miR-10 family members have been unveiled in *L. vannamei* (32). Based on the constructed *L. vannamei* miRNA libraries, the current study identified a novel sequence high homologous to miR-10 and miR-10a from other organisms. The two unconserved bases compared with other miR-10 homologs imply that miR-10c is a novel miRNA, which was further confirmed by stem-loop PCR, *in situ* hybridization, and in particular Northern blot, a gold standard for identification of miRNAs. Because the whole genome data of *L. vannamei* are incomplete currently (33), the genomic location of miR-10c has not been determined. In addition, it has been reported that the miRNAs in shrimp could undergo posttranscriptional edition (34). It is worthy of further study to investigate whether miR-10c is also a posttranscriptionally edited product of other miR-10 family members.

Toll/TLRs are a group of most important pattern recognition receptors (PRRs) that recognize invading pathogens to trigger innate immune response in both vertebrates and invertebrates (35, 36). So far, there are nine Toll receptors, named as Toll1–9 in chronological order of identification, found in *L. vannamei* genome. Toll4 had been extensively studied as an important recognition receptor to sense WSSV infection and transduce signals to activate nuclear translocation and phosphorylation of dorsal, ultimately activating the antiviral response in shrimp (37). It has also been reported that expression of Toll1 responded to the *V. alginolyticus* stimulation, and LvToll2 but not LvToll3 could significantly activate the promoters of NF-κB signaling pathway downstream AMP genes in S2 cells (12). The current study also demonstrated that there was no regulatory relationship between dorsal and miR-10c, suggesting that dorsal could not be a downstream transcription factor of the miR-10c-Toll3 signaling. A recent study showed that the *L. vannamei* Toll3 facilitated the transcription of IRF and its regulatory target genes Vago4/5 (11, 12). The IRF/Vago/JAK-STAT axis in shrimp is known to be similar to the interferon system of mammals and also play essential role in antiviral response (10). The connection of LvToll3 with IRF may indicate its involvement in regulation of this regulatory axis. However, the roles of LvToll3 in IRF pathway activation as well as in immune responses against WSSV infection are still unclear. This study further showed that silencing of LvToll3 *in vivo* significantly downregulated the protein level of IRF, confirming the regulatory effect of LvToll3 on the IRF pathway. Furthermore, the LvToll3-silenced shrimp were highly susceptible to WSSV infection compared with the control. These indicated that LvToll3 could enhance the antiviral immunity of shrimp *via* being involved in activation of the IRF/Vago/JAK-STAT axis.

In mammals, the miR-10 family consists of a series of well-studied miRNAs that have attracted more and more attentions because of their conservation and genomic position within the *Hox* clusters of developmental regulators (38, 39). The miR-10 family members are known to be implicated in development of various species, including mammals, fly, and worm, through targeting *Hox* genes (40). Some studies have found that miR-10 family members are de-regulated and play critical regulatory role in the progression of several types of cancers, which is generally related to Hox genes (30, 41). Although several shrimp Hox genes have been identified (**Supplementary File 3**), the localization of the Hox cluster in *L. vannamei* genome has not been determined because of the incomplement of the now available genomic data. The relationship between miR-10c functions and the shrimp Hox cluster is worthy of further investigation.

In contrast with that in other biological processes, the involvement of the miR-10 family in immunity was only concerned in few researches. In mice, the miR-10a targets Prdm1 gene to suppress the production of IL-10 in CD4^+^ T cells, playing important roles in regulation of intestinal homeostasis and in pathogenesis of autoimmune diseases (42). The parasites *Taenia solium* and *T. crassiceps* produce an abundance of miR-10-5p, which is important for establishment of immunosuppressive mechanisms in the host by acting on host cells to regulate expression of proinflammatory cytokines in macrophages M(IFN-γ) and anti-inflammatory cytokines in macrophages M(IL-4) (43). As the novel identified miRNA, miR-10c has a base different from known miR-10 and miR-10a in the seed region, and its targeting genes may be different from those of miR-10. The current study identified LvToll3 as a target of miR-10c, suggesting its involvement in innate immune responses. We showed that miR-10c could directly downregulate the expression of LvToll3 and also significantly influence the nuclear translocation of IRF. These indicated that miR-10c was involved in the regulation of the IRF/Vago/JAK-STAT regulatory axis and may play a role in immune response against virus infection. The supporting evidence was from the qPCR results showing that miR-10c exerted regulatory effects on expression of immune effector genes, such as several AMPs and C-type lectins with antiviral activities. More importantly, miR-10c could also regulate the expression of IRF and Vago 4/5, the central components of the IRF/Vago/JAK-STAT axis (13, 23), confirming the important role of miR-10c in the LvToll3-IRF signaling cascade. Further analyses demonstrated that miR-10c could promote WSSV infection in shrimp, which was consistent with the result of LvToll3 silencing *in vivo*. However, compared with the control, the effects of miR-10c on WSSV infection were abolished by silencing of LvToll3. These confirmed that miR-10c could negatively regulate the antiviral response in shrimp by targeting LvToll3. To further explore the role of miR-10c in immunity, other target genes of miR-10c require to be identified. In addition, it has been reported that the *L. vannmei* miR-10a could be annexed by WSSV to directly target the 5′-UTRs of vp26, vp28, and wssv102 genes of WSSV to enhance the translocation of viral proteins (44). To further explore the role of miR-10c in WSSV infection, whether miR-10c could target WSSV genes is also worth in-depth investigations.

Taken together, the current study identified a novel miR-10 family member in *L. vannamei*, a target of which was determined as Toll3 receptor. Through modulating the Toll3-IRF signaling, miR-10c could play a role in maintenance of immune homeostasis to avoid overactivation of immune responses. However, miR-10c also attenuated the antiviral defense to promote WSSV infection, which could contribute to the pathogenesis of white spot syndrome, thus may serve as a potential target for preventing WSSV infection.

## Data Availability Statement

The datasets presented in this study can be found in online repositories. The names of the repository/repositories and accession number(s) can be found below: https://www.ncbi.nlm.nih.gov/genbank/, MZ462071 https://www.ncbi.nlm.nih.gov/genbank/, MZ462069 https://www.ncbi.nlm.nih.gov/genbank/,MZ462070.

## Author Contributions

XX and JH supervised the overall project and designed the experiments. HZ wrote the manuscript, performed the experiments, and analyzed data with the help from XL, ML, LY, and SW. All authors contributed to the article and approved the submitted version.

## Funding

This work was funded by the National Natural Science Foundation of China under grant Nos. 31972823, 31772881, and 32073004; Natural Science Foundation of Guangdong Province, China 2020A1515011152 and 2021A1515010539; National Key Research and Development Program of China 2018YFD0900505; China Agriculture Research System CARS48; and Key-Area Research and Development Program of Guangdong Province, China 2019B020217001, Science and Technology Planning Project of Guangdong Province of China, 2018A050506027.

## Conflict of Interest

The authors declare that the research was conducted in the absence of any commercial or financial relationships that could be construed as a potential conflict of interest.

## Publisher’s Note

All claims expressed in this article are solely those of the authors and do not necessarily represent those of their affiliated organizations, or those of the publisher, the editors and the reviewers. Any product that may be evaluated in this article, or claim that may be made by its manufacturer, is not guaranteed or endorsed by the publisher.

## Supplementary Material

Supplementary File 1 Differentially expressed miRNAs after WSSV infection in *L. vannamei*.

Supplementary File 2 3′-UTR sequences of screened genes for miR-10 targets.

Supplementary File 3 Hox homologs identified in *L. vannamei*.

Supplementary Figure 1 Western blot analysis of the protein level of LvToll3 after PBS, WSSV, and *V. parahaemolticus* injections, respectively. The protein levels of LvToll3 were normalized to those of the internal control Histone H3. Each bar is mean ± SD of three independent quantification of the electrophoretic bands, ***p* < 0.01 and **p* < 0.05 by two-tailed unpaired Student’s *t*-test.

Supplementary Figure 2 Regulatory relationship between miR-10c and dorsal. **(A)** qRT-PCR analysis of the knockdown efficiency of dorsal. **(B)** Stem-loop qRT-PCR analysis of miR-10c expression in hemocyte and gill after the knockdown of dorsal. Values in the dsRNA-GFP control group were set as the baseline (1.0). Each bar represents the mean ± SD (*n* = 4), ***p* < 0.01 by two-tailed unpaired Student’s *t*-test. **(C)** Western blot analysis of dorsal expression in hemocyte and gill after treatment with miR-10c mimics and inhibitor in shrimp. The protein levels of dorsal protein were normalized to those of the internal control Histone H3. Each bar is mean ± SD of three independent quantifications of the bands, ***p* < 0.01 and **p* < 0.05 by two-tailed unpaired Student’s *t*-test.
