# Supplementary material for: miR-10c Facilitates White Spot Syndrome Virus Infection by Targeting Toll3 in Litopenaeus vannemei
Source: Front Immunol. 2021 Dec 7;12:733730. doi: 10.3389/fimmu.2021.733730 (PMC8688535; doi:10.3389/fimmu.2021.733730)
Supplement: Supplementary File 1 — Differentially expressed miRNAs after WSSV infection in L. vannamei. [file DataSheet_1.docx]

>bantam

TGAGATCATTGTGAAAGCTGATT

>bantam*

GATCATTGTGAAAGCTGA

>let-7

TGAGGTAGTAGGTTGTATAGTT

>let-7a

TGAGGTAGTAGGTTGCAT

>let-7b

TGAGGTAGTAGGTTGTTGG

>let-7c

TGAGGTAGTAGGTTGTATGGTT

>let-7d

AGAGGTAGTAGGTTGTATAGTT

>let-7e

TGAGGTAGGAGGTTGTATAGTT

>let-7f

TGAGGTAGTAGATTGTATAGTT

>let-7g

TGAGGTAGTAGTTTGTATAGTT

>let-7i

TGAGGTAGTAGGGTGGCTAGTT

>miR-1

TGGAATGTAAAGAAGTATGGAG

>miR-10

ACCCTGTAGATCCGAATTTGT

>miR-100

AACCCGTAGATCCGAACTTGT

>miR-1000

ATATTGTCCCGTCACAGCAGT

>miR-10*

AAATTCGGTTCTAGAGAGGTTT

>miR-1026

TGAGACAAGACTTAAGAAGCA

>miR-1028

TCGATTGTAGGTTAAAAGC

>miR-10-3p

CAAATTCGGTTCTAGAGAGGTTT

>miR-10c

ACCTTGTAGATCCGAACTTGTGT

>miR-1093

TGGAGGTCGTTGGCGAAGGA

>miR-10a

TACCCTGTAGATCCGAATTTGT

>miR-10a-5p

AACCCGTAGATCCGTAGTTGT

>miR-10b

TTACCCTGTAGAACCGAGCGAG

>miR-10d

AACCCGTAGAATTGAATTTGT

>miR-1122

AGAGAATCCGTATGTAGA

>miR-1168

ATTCACGGAAGGAGAAGA

>miR-1175

TGAGATTCAACTCCTCCAACT

>miR-1175-3p

TGAGATTCAACTCCTCCAACTTAG

>miR-12

TGAGTATTACATCAGGTACTGG

>miR-1226

TCACAGCCCTAGTTACCTAG

>miR-1247*

AGGGAACGTGAGCTGGGC

>miR-125a

TCCCTGAGACCCTTTCTTGTG

>miR-125a-5p

TCCCTGAGACCCTTTCTTGTGA

>miR-125b-5p

CCTGAGACCCTTTCTTGTGA

>miR-1270

TCTGGACGAGCAGGACGAGCAGTT

>miR-1281

TCTCTCTCTCTCTCTCTCCC

>miR-1282

TCGTTTGCCGTCTTCTGC

>miR-1306

AGGATTGGCTCTGGGGGTCG

>miR-1371

TAATGAGGACTTTCTAGAGA

>miR-1378

AGAAGGCTGGGTGTTCGGGTCA

>miR-1382

GCCGTGGTCGCGCTGTGGTC

>miR-1394

CCCTGGGGGGCTCGAGGGGT

>miR-13b

TATCACAGCCTACTTGACGAGTT

>miR-1-3p

TGGAATGTAAAGAAGTATGGT

>miR-141

TAACACTGTCAGGTAAAGATG

>miR-1498

ATAATGTAGATAAGGGAATTCGG

>miR-1503

CGAGGAAGACCGCGGCAA

>miR-156

TTGACAGAAGAGAGTGAGCAC

>miR-1566

CGGGACAAGGACTGGCTCTGA

>miR-157

TTGACAGAAGATAGAGAG

>miR-159

TTTGGATTGAAGGGAGCTCTA

>miR-1610

TGGCTTGTGAGAGGTACGGGG

>miR-1621*

GGTTCGCCGTAGAGCACCGCGC

>miR-1629

TACTGTCGGATGGGTTTGTT

>miR-1648*

TGGAGAGTTCAGCGAGCTGTC

>miR-1670

GGGGACGTAGCTCAAGTGGT

>miR-1692

GGATAGCTCAGTCGGTAGAG

>miR-172

TAGCACCACAGGATTCAGCA

>miR-1813

TTTGTGACTGCAAGGCTAGAG

>miR-1814c

TTTTTTTGGGGTTGTTTG

>miR-182

CTTGGCACTGGAAGAATTCACT

>miR-1832

TGGGCGGAGATTCGAAGAT

>miR-184

TGGACGGAGAACTGATAAGGGC

>miR-184*

CCTTATCATTTCGTCAGTCCCG

>miR-184b

TGGACGGAGAACTGATAAGGA

>miR-1858

GAGGAGGAGGAGGAGGGGGT

>miR-1889*

TAATCTCAAACTGTAAAGCTGA

>miR-1894-3p

GGAGAGGGAGAGGGAGAGGGAG

>miR-190

AGATATGTTTGATATTCTTGGTTG

>miR-191*

GCTCGTAGTTGGATTTCTG

>miR-1937a

TCATATCCCGGACGGGCCCCCA

>miR-1937b

ATTTCCCGGACCGGCCCCCA

>miR-1937c

ATTTCCCGGACAGGCCCCCA

>miR-1939

TCGATTCCCGGCCGATGCACCA

>miR-1955-3p

GAGCATAGATGCTGGGAC

>miR-1957

CAGTCGGTAGAGCATTAG

>miR-1959

GGGGATGTAGCTCAAGTGGTAGAGC

>miR-196c

TAGGTGTTTGTGTGTTGTG

>miR-1982*

TTGGGTGGGACTGGGAGG

>miR-1985

TGCCATTTTTATCAGTCACTGT

>miR-1c

TGGAATGTAAAGAAGTATTGA

>miR-2

TATCACAGCCAGCTTTGATGCGC

>miR-2001

TTGTGACCGTTATAATGGGC

>miR-206

TGGAATGTAAGGAAGTATGG

>miR-207

CTCTCTCTCTCTCTCTCCTCCCTC

>miR-2073

GCCGGGTGCCCTCCCCTCC

>miR-210*

AGACTGCAAGTGACATGCACAAGA

>miR-2123

TAAAAAGTCGACGGATCTCAAC

>miR-214

ACAGACAGACAGACAGACAG

>miR-216a

TAATCTCACGCGGTAAAGCTGAG

>miR-2261

TTTGGAGCTCAAGAACGAATGA

>miR-2284i

GAGAAGTTGTTTGGCGTAT

>miR-2305

GGAGGTGGGGGGAGGTGGG

>miR-2321

GGTCGGATGGTTTTGTTG

>miR-235

TATTGCACTTTCCCCGGCCTA

>miR-2354

TAGTAGGTTGTATAGTTAT

>miR-2356

TTTGGGATGATGGGCGTCTGAGGC

>miR-241

AGAGGTAGTGACGAAAAAT

>miR-244*

TAGATTTTGGGCTAAAGG

>miR-2462

AAAGGATTGGCTCTGAGGATTGAG

>miR-247

TGACTAGACTATTACTCATCT

>miR-2476

TCCCGTGTGGTCTAGTGGCCAG

>miR-2478

TCGTATCCCACTCCTGACACCA

>miR-2481

TACGGTCGGATGGGTTTT

>miR-2487

TTGGCTCTAAGGGCTGGGCCGGTCGG

>miR-2493*

ACACACACACACACACAGACACA

>miR-252

CTAAGTACTAGTGCCGCAGGAG

>miR-2527

TGATAGGATGTAGGTGTAAAG

>miR-252a

CTAAGTACTAGTGCCGCAGGAGA

>miR-2569*

CATGAACGAGGAATTTTC

>miR-263a

AATGGCACTGGAAGAATTCACGG

>miR-263b

CTTGGCACTGGAAGAATTCACAG

>miR-264

CGGTCGGATGGTTTTGTTATG

>miR-265

TGGGAGGAAGGGTTGTAG

>miR-2656

AAGAGCATAATCGGTAGG

>miR-2673

CCTCTTTCTTCTCTCTTCCTCAC

>miR-272

TCAGAAGGCTGGGTGTTTG

>miR-2720

TGACTAGAGAACTACTCATCC

>miR-275

TCAGGTACCTGATGTAGCGCGC

>miR-276

TAGGAACTTCATACCGTGCTC

>miR-2761*

CATCGAACGAGTTCCATCCG

>miR-2765

TTGGTAACTCCACCACCGTTGGC

>miR-276a

TAGGAACTTCATACCGTGCTCT

>miR-277a

TAAATGCATTGTCTGGTATGTCA

>miR-277c

TAAATGCATTGTCTGGTATGT

>miR-278

TCGGTGGGACTCTCGTCCGTTT

>miR-279

TGACTAGAGAACTACTCATCCA

>miR-279a

TGACTAGAGATTTCACACTCAT

>miR-279b

TGACTAGATTAGCACTCACCAT

>miR-279c

TGACTAGACTCCTACTCATCTG

>miR-279d

TGACTAGAGTCTCACTTATCC

>miR-2808d

CCGGACGAGAATCGGTGTGC

>miR-281-2*

AAGAGAGCTATCCGTCGACAGT

>miR-283

AAATATCAGCAGGTAATTTGGG

>miR-2840

ACGAACGTGGAAGAAGAGGAGT

>miR-286

TGACTAGAGAACTACTCATGCAT

>miR-2861

GGGGCCTGAGTGGCGCAGCGG

>miR-2868

TTGGTTTTGTTGGAGGAA

>miR-286a

TGACTAGAGAACTACTCCTCCT

>miR-286b

TGACTAGAGAACTACTCATCCC

>miR-2916

TTAGGGGATCAAAGACGATCAGAT

>miR-2944b*

TATCACAGTCATAGTTACCTAG

>miR-2950

TGGTCGTGCACGGAATGATGGAACA

>miR-2976

GCGGGAGCGGGAGGCGTGACGGGG

>miR-297b-3p

CATACATACACACATACACATA

>miR-2989

GCGCTTGAGAGAACTCTG

>miR-2993

CAGGCTGTGAGAAGGACGG

>miR-2a

TATCACAGCCAGCTTTGATGAGC

>miR-2b

TATCACAGCCACCTTTGATGAGC

>miR-2c

TCACAGCCAGCTTTGATGAGT

>miR-305

ATTGTACTTCATCAGGTGCTCGG

>miR-305*

CGGCATCTGTTGGAGTACATTAG

>miR-306

TCAGGTACTATGTGACTCTGC

>miR-307

TCACAACCTCCTTGAGTGAGTGA

>miR-307*

ACTCACTCAACTTGGATGTGA

>miR-3082-5p

AGAGTGTGTGTGTGTGTGT

>miR-3119

ATCGCTTTTAACTCTGATGGCT

>miR-3141

GAGGGCGCGGTGGGGAGC

>miR-315

TTTTGATTGTTGCTCAGAAGGC

>miR-316

TGTCTTTTTCTGCTTTGCTGCCG

>miR-316*

TGTCTTTTTCTGCTTTGCTGCT

>miR-317

TGAACACAGCTGGTGGTATCTCAGT

>miR-3178

GTAGGGCCGCGGCCGGATG

>miR-3201

GGGAGGTAGTGACGAAAAAT

>miR-3365

TGACTAGACTCTTACTCATCTGCAA

>miR-34

TGGCAGTGTGGTTAGCTGGTTGTG

>miR-3437

CGGTCGGATGGTTTTTTGT

>miR-3463

TCAGCGGGCTAAAGATAGGGG

>miR-352

GGTAGTAGGTTGTATAGTA

>miR-3558-3p

ACTGTCGGAGGGTTTTGT

>miR-3597-3p

CATAAAGCTAGATTACCAAA

>miR-3597-5p

TTGGTGATCTAGCTATATG

>miR-3650

AGCTGTGGACTGTAGATCC

>miR-375*

TTTGTTCGCCTGGCTCAGTCG

>miR-382

GAAGTTGTTCGTGGTGGATTCG

>miR-3946

CAGAGAGAAAGAGAAAGAGAGAC

>miR-395

CTGGAAGTCTGGAGGATTC

>miR-4000b-5p

TGAAACTTGAGTATAGGGGC

>miR-4001a-5p

TGGAATTATGGAACAGGAC

>miR-4001g-3p

TGTAACTTATTTTTGGACAA

>miR-4005b-3p

CAAGGGGAAGTCACGCGGCTGT

>miR-4006c-5p

TAGAACAATGTAGATAAGGG

>miR-4018a-5p

CGTGAAATTGTTGAAAGGG

>miR-4060-3p

GCTGTTGTTGAAGTGGAAGT

>miR-4077d-5p

AATGCTGGCTTGATTTGGACT

>miR-4090-3p

ACGGTCGGTTGGTTTTGTTG

>miR-4104-5p

ACGTGAAATTGTTGAAAGGG

>miR-4122-3p

TAAGTTTTGTTTGTATAGCA

>miR-4127-3p

AGGCGCTTGAGAGAACTCG

>miR-4171-5p

TGACTCTCTTAAGGTAGC

>miR-4175-3p

GGTGTAGCTCAGTGGTAGA

>miR-419

TGATGATGATGATGATGATG

>miR-4206-3p

GATTGAATGGTCTAGTGAGG

>miR-425-3p

CATCGGGGATCGTCAGCTTT

>miR-4259

CGTTGGTCTAGGGGTATGA

>miR-4260

TTTGGGTGCAGGAGTCCC

>miR-429

TGATCTGTCTGGTTAATTCCG

>miR-4383

TCATTGGATCTCTGCTGAACCGC

>miR-44

TGACTAGAGAACTATTCATCC

>miR-4413

GATGAGACTTGTAAGTACTG

>miR-466

CACACACATACACACACACACACA

>miR-466b-1*

CATACATACACACACACACACACACA

>miR-466b-2*

ATATATATACACACACACACACA

>miR-466d-3p

TATACATACACACACATA

>miR-466f-3p

CACACACACACACACACACACA

>miR-466g

AGACAGACACACACACAC

>miR-466h-3p

CACGCACACACACACACA

>miR-466i-3p

CACACACACACACATACACACACA

>miR-466i-5p

TGTGTGTGTGTGTGTGTGT

>miR-466m-3p

CACATACACACACACACACGCA

>miR-467a*

ACATGCATACATACACACACACA

>miR-467b*

ATACACACACACACACACACACACA

>miR-467f

ACATACACACTCACACACATACA

>miR-467g

CACACACACACACATATAT

>miR-482

GGAATGGGCTGTTTGGGA

>miR-552

ACACAGGTGGACTGGTAGAGCATA

>miR-571

TGAGTGTGAGCCTCTGAGCGG

>miR-574-3p

CACACATGCATGCACACACACACACA

>miR-574-5p

GTGTGTGTGTGTGTGTGTGTGTGTGT

>miR-575

CGCCACGTTGCGACAGGAGC

>miR-595

CCGTTAAGTTTGCTGTGGTGTGTCT

>miR-61

TGACTAGACTCTTACTCATCT

>miR-615

GGGAGGTCCGGAGCTCGGG

>miR-61b-3p

TGACTAGAGATTTCACACTTATCT

>miR-644

AGTTTGGCTTTGTAGAGC

>miR-650b

AGGAAGGCGCCTCTCGGGC

>miR-654-5p

TGGAGGCTGACACTGAACATGTGT

>miR-664-1*

TGGCTGTGGAGAAGATTGG

>miR-665

GCCAGGAGGTCTCGAGGCCCC

>miR-669c

CATGTGTGTGTGTGTGCATGTGTGT

>miR-669c*

CACACACACACACACATACA

>miR-669n

ATATATGTGTGTGTGTGTGTGT

>miR-669p*

CATACACACACACACACACGGA

>miR-7

TGGAAGACTAGTGATTTTGTTGTT

>miR-7*

CAAGAAATCACTAATCCTCCTA

>miR-702-5p

TGAGTGGGGGTCGTTGGCTG

>miR-709

GGAGGAGGAGGAGGAGGA

>miR-71

TGAAAGACATGGGTAGTGAGATGT

>miR-71*

TCTCACTACCTTGTCTTTCACG

>miR-71c

TGAAAGACATGGGTAGTGAGAT

>miR-739

AGGACTGACGTGGAGAAGGGTT

>miR-745

GAGCTGCCCAATGAAGGGCTGTT

>miR-745b

GAGCTGCCCAATGAAGGGCTGT

>miR-748

TTTGTCGAGAAGTGTAATGAG

>miR-750

CCAGATCTAACTCTTCCAGCTCA

>miR-762

GGGGGTGGGGTCCGAGGCCGAGGTC

>miR-79

ATAAAGCTAGATTACCAAAGTC

>miR-79-3p

TAAAGCTAGATTACCAAAGTC

>miR-8

TAATACTGTCAGGTAAAGATGT

>miR-8*

CATCTTACCGGACAGCATTAGA

>miR-84a

TGAGGCTAGATTATAAGAATAAGTGG

>miR-854

GAGGAGGGGGGAGGAGGAG

>miR-857

TTTGTTGTTGAAGGTGGATT

>miR-87

GTGAGCAAAGTTTCAGGTGTGT

>miR-87a

TGAGCAAAGCTTCAGGGGGTT

>miR-87b

GTGAGCAAAGTTTCAGGGGTGT

>miR-9

TCTTTGGTGATCTAGCTGTATG

>miR-900

TTTGTTCGTTGTACCTGGGATGC

>miR-908

ACCACATCCAATGAAGGCAGC

>miR-920

GGAGAGAGTGGAAGCAGT

>miR-92a

TATTGCACTTTCCCCGGCCTAT

>miR-92b

AATTGCACTAGTCCCGGCCTGC

>miR-92e-5p

CGGTGGGTGGTGGTGCATG

>miR-96

CTTGGCACTGGCGGAATAA

>miR-960

TGAGGATTGACAGATTGATAGC

>miR-965

TAAGCGTATGGCTTTTCCCCTC

>miR-967

AGAGAACTCTGGAGAAGGA

>miR-96a

CTTGGCACTGGCGGAATAATCAC

>miR-96b

ATTTGGCACTTGTGGAATAATC

>miR-98

TGAGGGAGTAAGTTGTATAGTT

>miR-98*

CTGTACAACTTGCTAACTTTCC

>miR-981

TTCGTTGTCGTCGAAACCTGC

>miR-995

TAGCACCACAGGATTCAGCATT

>miR-996

TGACTAGAGATTTCACACTCA

>miR-998

TAGCACCACAGGATTCAGCAT

>miR-99a

AACCCGTAGATCCGACCTTGT

>miR-99a*

CAAGCTCGATTCTATGGGGATA

>miR-9a

TCTTTGGTGATCTAGCTGTATGA

>miR-9b

TCTTTGGTGATCTAGCTGTATGC

>miR-9c

TCTTTGGTGGTCTAGCTGTG

>miR-9c*

TCTTTGGTGATCTAGCTG

>miR-H1

CCAGGGAGGGTGTCAGGC

>miR-H18-5p

ATCGAACTGGGGGGATTCAGT

>miR-H23

AGGAGTGGAGCTTGCGGC

>miR-J1-3p

TGTTGATCCTGCCAGTAGTC

>miR-K12-3

CACATCTAAGGACGGCAGCA

>miR-M1-11-3p

GTAACCTCGTGAAAGCTGTC
