# Supplementary material for: miR-10c Facilitates White Spot Syndrome Virus Infection by Targeting Toll3 in Litopenaeus vannemei
Source: Front Immunol. 2021 Dec 7;12:733730. doi: 10.3389/fimmu.2021.733730 (PMC8688535; doi:10.3389/fimmu.2021.733730)
Supplement: Supplementary File 2 — 3′-UTR sequences of screened genes for miR-10 targets. [file DataSheet_2.docx]

>JAK Genbank accession NO.: KP310054

TTGCAAGTAGAGATCTTTGGCTTAATTGAGTTGAGTGAGTTAGTGATTTTTTTTCTGTCCATTCAATTTACAGCTGTCAC

AACAGACCAACATTGGGACTACTGGTGTTATTGACTTTTTTTCCAGATATTTCTTACCAGATTACATAGTTTTCTGCAAT

TGGCAAAATAAGAAACAGTTTATTTCTTTTTAATATTAAGTCAGAAATATTGATTATACTTAATCTTGTATAGTCCATGC

TGTCAGTGTTAAGATGATAGTTAAGATGAAAATTATGTGTTGAAGTGTCATTTACATTTTGTATTCTGCCATGGATGTTG

CAGTCTCTCAGCTATAGTATTATTATTTTGTCTGAATACTGTAGCTTGTGTTTTGTGATACCCTCCAAAGAATTTTATAC

TGAATGTTTAGAAGATAGATACACTGTACAATATCAGGGGGAATGGCAGATACTTAGTTTCTTTTGCAATTTTGCAATTA

TGTCTTTGTAAATTTTTAGGTTTAAATTTGATTTTTTAAAGATAAAAAACATTACCAATATAAGGTGCAATGATTTTATA

ACTTTCATAGAATTTACTAATTTGGTGTTATATTTCCTACTTATTGTATCTTAGAAATGTTCTATGGTATTGAAGATCTT

TCCTGTGTAGAGGATGCTGGTCAGCGGGCTTATTTTCTTGTAAAGTACGGCTTTGTGTGTATGCATTATCTCAACTCTAT

TTTCATAAAAGACATTGCTAGAATATTTCTGATACTGCAGTAACACCATACTGCCAAAACAAAATTATTTTTTGCAATTT

ATGATGATTTTTGGGGGATATGTGCTGAGGATTTAGACTAGAGTCCTTGGATGAGTAAAGATGATGGTCAGATTATAGTA

ATCAAATATATATTTATGAATAGCTTTAATTGCCTCATGCTTTACACAGAGTGGGGATATATATGATAGAATGTTCAAAG

GCATATGTGGAACACAAATGTATGTATGTTAATGTAATAAAATACATTCCTACACATGGCATGCAAATGCAGATATTGTG

ATTTTATGTTATTGTCACTCTTATGGTGTAAATATGTTTAGTATATTGGCTGAAGTCCGTAAAGAAGTTGTATCTAATTT

TCTATACATCCTATTAAATAATATTTTGGAGTCATGTGAAATTCTCATTGCAAATAAAGTTGCAATTTTATAATATTTCA

TGCACATTATGAATAATTTCTTTTACTGGTAAATTTTAATATATTTGACGATAGGAGTCTGATATTTTTATGCTGATATT

TAATCCAAAAGATACATAACTGTTTTATATGCAGTAGGACCTGGATATACGAATGTCTTACATGACATTCTTGTTTTGCA

CTCACATCTGAAGAGCCAGTGGAACAGAAATTGTAATTTGCTACTAGAGTGCAGTTGGGAATTATATATGTAATATATAA

TTATAATTATAAAAATATGTAGCACCTTGTAGGTGCTACAAGGGTAGATTAAAAGACAGCCAGCATGCTAACCTTCCTGT

GATAAACATGTTTAACCCACATCCATCTGACAGCTGCAGGAAGGGGATAGGAAATGACAGGAGACTTTTATCATTGCTAT

AATGTATTGAGCAGATATGAGCCAGTTATTTACATGTGGCAAAACAGACTATCAGATAATCTGAAAAAGTTAAGATTCTG

AGATATATATAGGTGTGTGAGGGTTGAATAGAGTAAAATGATGAATAAATAGGGAAACTCTTTCACTTTCTTATTTATCT

TTGTATGTTATACCATTTTCGAACAACAGCAACAGCAGCAGCAACAGTGACAATAACCAACCACAACAACACAAAAAAAA

AAAAAAAAAAAAAAAAAAAAAA

>STAT KC779541

AGTTTGGAAAAATGTTCACCTGCATTTGTAATAGAAAATGTTTGTTAAGATATATACATATATATTTTTAACAGAAAGGA

TTGAATTTTGAGGAAATATAAATCAAAAGAAAGGAATTTTTGTGGGGCAGGGGGGTCTAGTGATTTATTGAAATCATTGT

GAAATTTTTACTCTTGAAAGTTTTTCAATTTATCTCCAGAAGAGAAAGCTGATGAAAATAAAAATAGATACATAGAGAAA

ATATGGGAAAAAAAGGAAAAGAAAAAAGTATGGACAAGGTGTATCTTGATAGTAAGGTTTCTTATATTATAATGATTTTA

TTAAGCATAAAGATTACTTGTGGTATTACATATTAGAATCATACTGCATATATCTTCCCTTTAAGATTATTGTATTTATG

TCATATATGAAGACCCTGCCCAGTGTTTGGAAGATGAACAATTATCTGAATGATTTTGCCTCTAATTGTATGGAGTAGAT

GGAGTATCAAGTTAATGCTTAAGAGATTATGATTATTGTCATTATTTAGATATTTACTTTGTTTTAGTTTTTGTGTTTTT

TGTTTTTTTCTTTTCTTTTCTTTT

>IKKE JN180644

AATGTGTGAAGCTCAAGAAGAAGTTATGAGACTGCTGGAGGAAAATGGGACAATTATCAAGCGATTCCAGCGCATTACAA

GTCAGTCTGATCTATCGGAAGAGCATTAGGGAAACAAGCATTATTAGTGAGATTCTTTTGTTATAATGTTTTAGGCAACA

TCTGCAAGCACTTGATTAATTTGTTTATTTGCTTATTTTTCTTACTCAAGTTTTCAAGGTCAGTGGCTTATATTATTGTA

CTTACCATAAAGTGTTTCAAGTTAAAAAAAAAAAAAAAAAAAAAAAAAAAA

>IRF KM277954

ACGAACTCAAATTAATCATCATTTTTGTATATAACGATAGCTTTTTTTTGCGGTTTAAATATTTGTATAATTATATGTAA

ATATATCTATATCTACAGCAAAAAAAAAAAAAAAAAAAAAAAAAA

>DOME KC346866

GCATGTATCATTTCTAGTGAAGTTTTACTCCATTGTTGCATAAGAAATGTTTCAAGTCAGTCCACTGTTATTCAATATAT

TTGGCTTGAAGAACATATAGAAGTCATAAAATAATTCAGTTATGTGTAAGACTCTTCCTGAGGCAAAGTCCAAAGGCAAA

GTTTCCCTTAATCTGAGAAACTTACTTAACAGACTTCCATAATACTGGATATCAAATGATATATGTAAAAGCACTTTATT

GTGTGTTACCAAGTATTTATGAATGCAGTGGTTCACTCAATATTCTGGAGCATAAGATGATGAGAAGCAGCCAATATGTG

CGTCTATTATTTTGTGTAGCATTGTTAATTGGGGGGGAGGATTTTAGTGCAAATCATGGTGGTGTTACTAATTCTTGATC

CCTCAGTTACGTACGTGGTACACCTTTAGAAAGTACAAAGATATGATCCCGGTAGTGGTGTTTGCCCAGCAGTATGTGAT

GGTATTAGTTATGTTAAGTTGGTGTATATATGGATAGAGTTGTTAAAATAAAAAACAAAAAAAGAAAATATGTGGTTTTC

ACCATTAAGGTAAAAGAAGTCTTCAGAAGGCAAAAAAAAAAAAAAAAAAAAAAAAAAA

>SOCS2 KX174314

CCCCGTGGAGTTGCCCTCAAGCAGCCGTCCTTGACTTCGCAGAATGATTAGGGGGCAGCGGACAGGCGTCGGGCTCGGCG

CTCGTCATACAAAGGGAACCGCTGGCCTCCCGACGCATTCTTTGGGTGCCAAAAAGGAGAAAATAAAACTTGACACTTTA

TTATTTAAGAAAACATGTGCGACTGTGATCAATTTTACTCGTGGTAGCTATTTCAAGGACCAACTCTTCCTTACACTCCT

CTCCTGGGCCGCAGGGCTCCAGGGCACGCGATAGGCTAGCCAGAAGACTTTGCTCACGTTCCTCGACCTGGGAGGCGTTG

GTGAAGGGCGCTGTGTACGAACCTTTCTGGAAAAAGTGGAAAAGGATGATAAAATGAAAGTGGTTATTTCTATGATCTCA

AAGAAACCAAACGTTTTGGTGCGAAAAAGATTCTTGTAAAAGTGATGATTCTTTCGATGTTGATGATGTCCTCTTTTTGT

TTTGATTATTTTACTTCCATGCTGTAAGGAGTGC

>AGO2 HM234690

CAAGAATTTGCAAGCCTGACCAGATCCAGATAGTGTCTCTGAACTTTGCTGTTCATAATGAAATGCACTTCTTGTAGCAA

GATTTTG

>DICER2 HQ541163

TTGTTTCTTCTGTATTTGCTTTATATTTTACTCATTAATGAGGATTGCATGATCTGCATGTTTAATTGTAGGATTTTTTT

TTTTTTTTTTTTTTTTTAATAATATATTTTTAAAATGACAATGGAAGCCATTAGTTTTAGGTTATCCTGCTTAGCAAAAG

TGATCAATCAGGCAACTTTGACATATATATGGGTAATAATTTTCAGGCTTAGTGTTTAGTTAATTGTATGTATGTGTTTT

TTTTTTTTTTTTTTTTTTTTTTTTTGCCATCTAGGAAGTGGTTCTTCCCTCTGCATCTGTAGGTAACCCCCATGGGTTTC

AAGGGATTATAGGGAGGGGAGGGCTGTCAATAAGTATAGGATACAGGAACAAGGAAGGTTATGTTGAATATAAAGGACAA

AAAGTAAGCAAAGGCTTTTACATAATGTGAATTGTTAGCAAATGCACTAAAGAAAATAGCTGAGATAATTACTTTATCTT

TTTTTTCCACAATGCCTAGTAATATCCCTTCTGGATAGGGAGTATACCCCCAGGTTAAGAACCGTTGCTTTAGTGTTGGA

AAGTGTCATGGTTAAGCATCAGTGTTAGAATGGTTATAGGAATTGTTGGCTTTTTACAGGCCATTTGGAAAATTTAGGTA

AGGGAAGAAATTGAAATTGTTTAATAAAAAGAAAGAATATAAAATTGTTTAAGATAAGTTTTGAATATTTTTAATATTAA

GGAAGTAGGTTTATTAGGAAAGTATTTCACTGAGCTGTTTATACATACTATTGAAAAGTGTCTGCAAGAGTTTACCTGAA

GTCTGTATCTATGAATACACATCAATTTTAAAAGAAAAAAAAGTCTCCAAAGCACAAAAAAAAAAAAAAAAAAAAAAAAA

A

>VAGO1 HQ541158

GTTACTCCAGAAAGACGAGGCAAAATAAACTGTAATGATAATAAAGTTATTTGGATAATAAAAAAAAAAAAAAAAAAAAA

AAAAAA

>VAGO2 HQ541159

ACGAAGACGAAGGTTTAAACAAAGGGAAATGTTTATCGCAGTTTTTTTTCTCTCCTTTTCTTTGTTTCGTTTGTAATTCT

ATTTCTTTTCAGTGGGGTAAGAATAAAAAAGATTGATCAAGATGGGCCTTTTAATGTTTCGTGGAATTTTGTGTGTGTGT

TTTCTTGACTCTTAACATTTTTTCTGATTTGTTAAATATAGTTAGAGCTTCCCTATAAAAAAAAAAAAAAAAAAAAAAAA

AAA

>VAGO3 HQ541160

TCGAAGACGAAGGATTGAACAGAGAAAAAGAAAATGATGTTTATCATTTTCAAAAATCACATAAAAAATCGTGATGTTTA

TCGCAGTTATTTCTTTCTTTGTGTTTCGTTTCTAGTTTTATTTCTTTTCAGTGGGGTTGGAATAAAAAGATTGATCAAGA

AAAAAAAAAAAAAAGACACAAAGAAATAATATTTTAAAAAGGAGAAGAAAATTAGCGAAAAATAGAAAATTGCAGTACTC

GCAAATCCCATAATATTCATTCAATTTTTTCCCCTCTTTTTTCTCTCTCGCTCTTCCCCCTTTATCTAAATATACTCATT

TTCTTTCTCCTGTGAAAGACAAACACAGTCGGACAAAATAGAAAAAAAGAGATAGGGGGCGGGGATTGGGGGAGTTTGGG

>VAGO4 HQ541161

GGAGGGACTCGGCTGGAGAGTCCGGTTCTCGCGTCCGCACTTGACCAAAGCTTTTATTTTATCTTTTTTTTTAATATCTT

TTGTGTGTTCTTAGGCATTTATATTTTCCCGAGAGAGTTCGCTTGCGTTTTGGCCTCGCTTTGTTAACTGGACTCTTGAG

GTAAGATGCCGTTGTGAAGATCAGTTGGAAATTTGAAATTATGATGCAGAATCTTTGGGAGACGAAGGAATCCAAGGCTA

GCATTCCTTGGCAAAGGGAGCCAATGATTGATTTATTAAGATGTTTTTTTCCAAATGTATTTATTAAAAAAGAAAAAAAA

AAAAAAAAAAAA

>VAGO5 HQ541162

GAAATTCTGAGTTGTACCCCAATGACTGCTGTTATCCTTACTAATTCATACCTTATTTAGTGAAGCTCCTTTACATATAC

CTCCATTAATATGTTAAACACATGTTCAGCATTGTATCATGGATGAAGGCAATATATATGAAATGTATAATATTGATATT

GGGGAAGAAAAAAAACATTATAAAGAGTTAAAAAAAAAAAAAAAAAAAAAAAAAAAAAA

>PIAS MK987066

AATCCTAGCCAAATATATTTGTAGAGAATTGGGCAGGACCTTTGAGGGGATGTAACGATGGTTGCGGCATTTGCGCATCA

CCACTACCACTACTACCACCACCAACAGGGTTAAGGCACCTCAGCAATTTGGGAATAGCAGGAGCAAAGTTTCTTAGACA

AACCATTTCTTGTCTTTGGAAAATAATACATACTCTTCTTTTTTGCAAAATACCTTTTGCAAAACTTTCCTTCACCAAAG

TGTTACAATGTATTCTTAGTTGAGTGTAGAGGTTATCTTCTCATAGTCTTTTGGTTCTTGCAAAAGACTTAATAGTAGTT

GTAAGTAATAATATTGGAAATAGAGGAAATGTTACAAGTACAAATGTATCCTGTGAGAGTTTGTTCCTTGAGGGATCTGG

TTAACAGCTGTACAGTGAGGAGAATTGGACTAACGTGAACATTTAGATTCATTGGTATTTCACATCATCTTTTGTAACTT

CTGTTCTTCTTTGTGTCCACTTCATTTTTTACCTCAAGTTTGCCATAGTTTTATGTAAGAGATCTTTATCTTTACCTATA

CCATTTCTTTCTAGTGTTTATCACTGAGGCAGAAGTTTTACTGGCTTATTGAGTCGCATAAAAAAATCCATTTCTATTGA

CTCATGGCCTTTGAGTTATTTTTATCTTACTGAAAGTTATTTACACTACTTGTTTGTGGCATTAAAGTGGCCTCATTTAG

TCAAAGGTTGCCCCACCTTTGGACCCAACAGGGTGTGTGTGGTAAGATGGTCGGATTCCGAGGTAAACAACAACGCATCC

ATTGGTCCCCTCAAGTATGTGGCAACCACTAACGTCCTCTCCAGTAGCAGTAACTCCTGGAGCCCATCTCAAAACTTAGT

CGTGTCCACCAGAAGTGAGAGTCCCCTACCCCAAGAGGGAAATGACTCCAGCAGAATTCTCGCCACAAACACATGCTACC

GTAAAACATACAATCTCAGGAAAAGGTCCTCAAAAACCGCTGCCGAGTGATTACATTTTGAGGAGCTGTGATTCTTCATT

TCTTTTGATAATGTTGATGCCTCATCAGCATTTTTTTTTTATCAATCTTTATTTGTTCTTTCTTTCATTCTTATTGATTT

TCTTTTCTTATTTTTTAACATGACACTATAGTAAATCTTAGTTTTCTATAATGCATTTACTGCCAGCAAGATAAACATTT

TGTCTTCTACTTGAATTATCAAGTGCATCTATTTGTTCTGTTCTTGCCTCTAAACTGTACAGATGTTAACCAAAGCAATT

TGAAGAAATGTGTATATATATTGTGTATCAACTATGAATCTGAAGTATTTGTTAAAAAAAAAGTTATGCTGCAGCTTCCA

TGTGTTTCATTGTATGATATATTTGTCTAAGCGTATCAGCCTGACCATGTAGTGAATGTGTATTTTTTTTTTCAATGTAG

TGTACACATAGATTGAACACTACTGCTCAGTGATATTGAGACAGTAATAGGGTATATATCTATGTGTTTATGATTTGGAA

AGAGTGAAGAATGGGGAGGGAGGATAATGATTTTTAAGGATATCTGCAGTATAGATTTTTTTTTCTTGAATTATTTATTA

TGTAATAAAGGATAGACATTGATTTATTGTTACAATGAAGTAATAAGAATTAGTTTTCTTTTCTGTGCTTTCTGCAAATG

CAGACGGTTATACTTCACACGGCTATTGTCATTCTCCTTGTTTACCCAGCCTTCCATATGTCCATGACACTACTCTGCAT

GGCTGATTTTTTTTAC

>TOLL3 JN180638

AGATCCTGCGGAACTCCCTGGTAGTCCTGTGGTACTCCAATGGCGACCCTTAGTCAAAGAGAGCTTTGTCGAAGGGCTCA

GTCCTAGGTAGAGCTCTGAGATTATGCAACACGGCTGTAGTTTTACAAGGGCTCTGTGATGAAAGCTCCTGGCTGTGGTT

ATAACGAAAAGGGCTTTAGTGAGAAGGATACATAGCTTTAAAGTGACACATGTGGTTATAATGGACACGAAACGGAAGTG

TAAGAATCGAGCTTGTGATGCAATGTTTTCGAAGTGTAGTGGAAGCAGGACCGTTTGTGGACGATGGAGCCATGTACATG

TTATAGCAATCTCCATCGTTGAATATGTAACATCTAGTCACATATATATTTATATATATTGTATATGAAAGTGAACAGTG

ACTTGGCTTTCACTCCGTCATGTATATGGAAAAAAAACAACGAGCCGACAACTTTTTATGGCAAATATCTACCGTTAAAA

AAAGCTTTTTTGTAAATGTGCGTGAAGGAATATGAAAAACAAACAAAACAAAAATACCCGCAGTATCTATTTGGCCTCAG

CTGTTATTGTCATAAAGACATGATAAATAAAACTTTTTTGATGCATAAATATGTTGTGCTATGGATGTGATCATTGGTGC

ATGCAGCCAGTTATTTATTCAGACTATGCTTTATGGATATCGTTCACATGTGTTTTATTGACATATGGGAAATGTATCGT

CACTTCAAACAGAGATCATGTTTATGTTATTGTTTATCTCTGTAATGTGTAACCATTGTCACGTCGTTTGAAAAAGACTT

TTGTTAAAATCTGTTTGAAAAAAAAAAAAAAAAAAAAAAAAAAAAAAAA

>Spz1 JN180646

tacgccaccagaatgctcttacaggaatgagatcttagactggatctaggactccctggccacgacggaaccaccgacaa

tccctataaccccgtccctgaaagcgcaggagaagacccgagatgactggagaactgctttcttcacgtaccgccactag

tcatgccaggaactcccgggacgctcccgccgctgctctcacttttccttttctttttcttctttttatatcccatgtac

atggtagatacgaaaagtctaatgtaatattttttatgaataaattattttgaattataaaaaaaaaaaaaaaaaaaaaa

aaaaaaaa

>Spz2 JN180647

agtgtgttgtcgaggactttatctgctggtgaaacgtttcgttgggcacttcatctacttttgaagcgttttatcgaggg

ctctgtatagtgctagagagattcgttgagggtttttatctacttctgaagcggtatgtctacggttttatctgcttcag

aaacacttccttgaggtgtctgaagcgtcttaccgaggaattaatctacttctgaagcgtcttaccgaggaattaatcta

cttctgaagcgtcttaccgaggaattaatctacttctgaagcgtcttaccgaggaattaatctacttctgaagcgtttcg

ttaagggctctatctactactgaagcgtctcctgaagctccgaaggactgaaggaatcgctgatgcttgacattttaact

cgtgcttttgtcgtcggtgtgctgcagggagggtcgccgtagttatagatattgttgtttgttataacttgtgttggttg

agtatggttgattattgtactttttttatcggttgaagagggatatcttttgtttttgctttgcatgttgtaggttctat

tgggatgttactttattattatttttatcggttaaagagggattttattttttgctttacatagaggtttaactgggttg

ttacagtttttagaaattcagatgaggagggatatttctgattttgtttaacttgttgtcaatggagtcttttgatatct

tgaataaactttcgtgtaatgattataaaaaaaaaaaaaaaaaaaaaaaaaaaaaaa

>Spz3 JN180648

ccgcgaagccataaatcggaagccacatttaatggggtaaatgacctcatcggcgtgagcaatgttttaatttttctaac

gacctggtaaaagtagcggaaggcataccatccctggaatgacttaaaatacgtgaagagagcagttctct

>Toll KT372179

cgcaagttgaacccaaaacttttttttccgtgtacatgagtagctggactactgtcttcacagtgatgactcgttcaaag

aaagtgttccagatatgaaaacatatatagacagatgaatatatatttatttagaaaattatatggacttattcccaaca

gttcctcagatagtaggaatgtggatataaatgttgtatgcagctaaacttcttacaacattgactttgtaccattggtt

gtaatttgccaattggctgtgacctgtattgtaaaccaggtacatatgtatatagcaggtttatgaaaaaaaaaaaaaaa

aa

>Toll-i JN185616

acctgactatcctatagtcatcacattatttccaaagtatagttgatacataattgaaaaggtggaaatttgtaaaatca

catttatctctcccttaatgtatatccataattttgttttttgaaaaagtatcacataatgttgatgtttaattgcaaga

actatatggtactccaatcatattgtctttaaccaagggtgtataatcattatatttatgtttgcttttaaactataagt

aatgtatgagataattatccatctgttttacacttccttcatatatattggatgtttatgaaaaaaaaaaaaaaaaaaaa

aaaaaaaaaa

>Toll2 JN180637

aaattaacttaatgaaaaagcacattggagttcataagcagagtgtggaagtggcaaattccttttagacttgaaaatta

gtttctgcttacaaactttatttactcaaaagtactaattactgtcatcattatcagtgatacctctaattgtacggatt

gcaaaaaaaaaaaaaaaaaaaaaaaaaaaaaa

>Toll3 JN180638

agatcctgcggaactccctggtagtcctgtggtactccaatggcgacccttagtcaaagagagctttgtcgaagggctca

gtcctaggtagagctctgagattatgcaacacggctgtagttttacaagggctctgtgatgaaagctcctggctgtggtt

ataacgaaaagggctttagtgagaaggatacatagctttaaagtgacacatgtggttataatggacacgaaacggaagtg

taagaatcgagcttgtgatgcaatgttttcgaagtgtagtggaagcaggaccgtttgtggacgatggagccatgtacatg

ttatagcaatctccatcgttgaatatgtaacatctagtcacatatatatttatatatattgtatatgaaagtgaacagtg

acttggctttcactccgtcatgtatatggaaaaaaaacaacgagccgacaactttttatggcaaatatctaccgttaaaa

aaagcttttttgtaaatgtgcgtgaaggaatatgaaaaacaaacaaaacaaaaatacccgcagtatctatttggcctcag

ctgttattgtcataaagacatgataaataaaacttttttgatgcataaatatgttgtgctatggatgtgatcattggtgc

atgcagccagttatttattcagactatgctttatggatatcgttcacatgtgttttattgacatatgggaaatgtatcgt

cacttcaaacagagatcatgtttatgttattgtttatctctgtaatgtgtaaccattgtcacgtcgtttgaaaaagactt

ttgttaaaatctgtttgaaaaaaaaaaaaaaaaaaaaaaaaaaaaaaaa

>TRAF6 HM581680

ttagaatttaacaaattgaaaaatattttttaatcatttggatattaaccctgtggtgatggatttttcttacaaggctc

ataccagggtgcatgtgatatcagcttcttaagtgaaaaattagaagtgcagggagtctttatttactccactctttgtg

tgtttggctgcagcagatatgatttattagaaactgaggctaaaacaaggctttaacccaacttttgagggattgtaatt

tgattgcctcaatcaagagccaaatagcaaacagctgtttaggtgcacaacttagagtagctttaccatgcatggtgtgg

atccctggcacccaccatttgggcctggtgtgtaatggtaagaatggatgttgaccattaagaagggtttagggagggtt

gtgatgaggaatgtggaagatatttttttatacacaatagaaaatgaatcatcaagaagttttaagaagtatattttgga

ttagaatttaattattaaattattattgttgttatcatcataatccacatttttgtcattgtagtaattaattatttttg

ctattgagaagattgttattgttatatagtttttatattaatgttttttatatttgaaatggtatcataatttcctactc

ggaatatactgaatgtctgtaatggagagagtagtgtaaagattttacagagcattccattgttactgcagtttgtttat

aaagcttctgtgcaaaaaaaaaaaaaaaaaaaaaaaaaaaa

>LGBP EU102286

atccctccactcaactccactcaacaaatcaaatgttatttaactgtctttatcacgttttttattgttattatcgccat

gtcatcagcaaataaataaaaatgaaagcatttacaataaagatattcattagaaaaaaaaaaaaaaaaaaaaaaaa

>MyD88 JX073568

tttattttacatttgctttgcacaattttactatgataatgttttaattatttttgactgataaggatgactggagcatc

tatatactaaggttgtacagaatgatagaagagtgttttaacgtttttcaacgacaattataaatcgcttgctttcatat

tacatatattgtttattatgggaattattttgaatttagatgctcctgctctgaggacaaaggaagaaatgtctcatata

tttcataattttacttgttcatgggctacactttgccaatttgtagattgaacttgtatatcactctctagtagcttata

tattcatgtttttaattatctctgacacatacagtttagaacattctcggtgtggacagcagtatttgttttacataatt

ttacaaaaagtcctcatacttttttgtactagttgcctgaagatactaataacagttattttaagattattatttagcta

caacaagtctatcagtaaaggatatttaatactgaatttgaatgctggcaagattattatgtgtagaattgtaattaatg

tgtttgttagcaaatgtggaagatatttatttgaattcataccttttctacattaatatcatttttatatttttataagg

caatgcagggttttcctaagcagtttctgcatttataggtcaataaagacataaatgattaggtataagatgacatatta

ctgtaaaataaatatgtgcatgatgcagaaaaaatatatttttaaggttaatctttgatatgattgacactcgagaggaaaaaaaaaaaaaaaaa

>Pelle JN180645

tgtacacagtcagtaaaatttagatgcactaaaaaaaaaaaaaaaaaaaaaaaaaaaaaaa

>Tube KC346864

TTTGCAGGAAACTCATTCTTTACTGTTCAAATTCAGCACTTATGCAGTGCACAAATCTAGATTTATGAAGCTTTTTGACA

AATTTGAAATATTATTTATATATAAGAATTTCTTGTTTTCATAGACATTAGACATTCTAGACATAGAAGTTTATATTAAA

AAAATTTATTTATAAAAGAAAAATCTTTTATGTTGGTTTCAAATATATAATCTGTAAAGCTAGGGATCTTTGTGGAACAT

GAAGAGGTTTTCTCTTGAAAGACATCAGATATTGAGCAGATATATGTACAGCAACTGTTTTTCAGTTATGGCCCTGTTTT

CTTCCTGTTAGTTCAATACACACGTTAGCTACTTGATGAAAAAGGAAGATATTAATGCATGTTACTTTTAAATTGCATTA

CTCTTAAAAGCTGACTTTTGAATATTAGAATTTTAGGTTATCAGGTAAACATATCCTCCTACCTGTTACATTCATGTACT

TTTTTATGCATAATTAGCATTAAGAAAGATTAGAATCTGTCAATCAACTGCTAAAGCTACCATAGCCTGCCTAACTGCCT

AGTTCATCTTATTTAGAAACCTAAATCTTGGAAGGGCAGATGCACCTTCTCTCGAGTTTGCTTTTCAAAACATAACTAGG

CTTTCTTAGTCATGAACTTAGTTGTTTTGTTATATGACCTAGATGTCTTGTCTTCACATTTATATTTTTGTAATTGTTCT

AGGTACACACTAAGTTCAGTTTTATATTCATTAACAGTAATTGCTTTTGGAAGGGATGGAAACCTCTCTCCTTTATAGAT

TTTGAGAAATATGCTGAGTCATCTTGTTACTGGATTGTGTAAAGTAATTTCTTCATATATGTATGTATAGCTGCATGTAG

CTTTACATCTGCAATGTATAGAAACTGATCAGTGCATTTTATACCCTTTATATACAGTTTGTTATACTTTTATGTATATT

CATTTTGTGCAGGTTACAATTTTTTTTTTCTTTTTTTTGTCATAAGTTGTTAGATCAGTCTGCTAAAATTTTTGAGATAA

AGGGCTTCCTCACCTTTTCTTTGATAAATGCGCAATGTTCTTGTGTCAAAAGGTGCTCCCTTGATTTTTGGTTTGATTAA

TTGTACAGCAGTGGTCATGTTAAATGATCTGTTATGGAACTTTCATTTGTTGTAACCGAGACAAGATTTATACTAGTATT

TTACTCCTTGGTGAATTTATTTACAAAGTAGTAAGAAAAGTGGAACTATTAGTGGAAAATAAAATAGGTATAGCAAGAGA

GAGCATGGTGATGCAGTGTGTTGCAAAAGGGGTTGGGGTAGTACAAATAATATTAAATTAGGACCTTTGACTTCATTAGA

AGCTTCTTTTTATACTCTTCCCTCACTTGACTAGGCCTTTGGTGACATTCTCATGCTGCATCTTACATTATGCTTGTATT

GTGTGGTGGAAAATAATATTGCATCACAAGATCTGTTTCTTTGACATTTGCTTTCACCTAACGCTTGTAAAAGGAAATTA

ATGATTTGTGAATAAATTATAATAAACTCTGTTATAGTTCCTTCAGTTCTACTAATAAGATACTGGAGTGCCATTTTCAT

CAGAGAATGAAGATTGCTGCACCTACTGATTATGCTTCTGTAAGGAAGAGTGACTGATGTAATATTATGATTTGAAGTGG

TTATAGAAACGGAAAAAATCCGTGCCGTTTGGCCTGAAAATCATCTTGTAGAAACGGATCTGTTTCTCTATCTGCAGCAA

ATCTTTCTGCAGAGACAGGCAACTGGAACACTTCCTCAAATCGAAATCAAGGTTGTCGAAGGAAGCGGAGTACTGCAGAA

GATGACCTGCATTGATTAGGTTAGGTTAGATTAGTTTGGTTA

>Cactus JX014314

GAAATGTTTGCGTACCCATCAGTAGCTGGGAACTTACAACTTCTGATGTGATTTTTTAAGATACCTGTAACAGGCATCTA

GTATCAGTGACCTGAGAACCTTCACAGCGGCTGGAGTGCAGGTCCTGTTAAATTGTTGAATGATGATATTCAGAAAAGGA

TAAAGTGAAAGACCTTTTTGAAAGTAGTGTCAGTTTGAGGAAGACATGTCTTGTTTGGAGATAGTGTCAGTGTGTGACTT

TGAGGAGTCAGGAGTAATGCTGGTGCCTTGGACCTACCTGACCCTGTATCAAGTGATTGTCAACAAGGGGAGTCATTCCC

TCGCCTGCGTATCATCATCATCAAAGAATACATTAAGTGGAGTGGGAAGCTGTACCCAAGACAAAATATTGACATTTAGA

CAACCTTGCAAATTATTCTTAGGACTTGTTTTTGAAAAGATCTCTATGTTGAAGAAGAAATTGAACCCTTGCTTTATTAA

AAGTAGTGGTCATAATATTAATTTCTCAATACAGTGTGTGTATGATCGTCTACCTGTGATCTCATATAAAAAGGGTTGGT

GTGAAGCAAGCTGGGAAATGTGTAAAAACTAACACAGATACCTGTGCATTAAATTTTGTGTTTCATCAGTTTCCTGCAAA

GGAAATACTAGTATACTTGTTAATAACATTTATGTAGTTAAGTTGTATTGTATCTAACTTTGCACTTTCCATTGAATATC

TTAATAAGGGTGTAAAGGAACATTGTGATAATACAAATGTCCTGTTGCAACCACAAAATCTGGGGAACCAGATACCTGCA

GGCTGTAGCTGCACTCATTATTATTATACTGTGAGAAATGTGTTGAACCAGACTGATACTTGCTCCTGTCTTGCTAGTAT

GAGGAAGAGAAATTATTAGATGTTATATACACAATGTTCTTTGTTTGGCGTTTTGAATAGCAGCATCAGGTTATCTGGCT

GTAGATGTTGGAAAGTTACAGCTGCAGTCATTGTTGTTATCTCAATAATTAAATACAGCAGTGAACTGCAGTATTTGGTT

GAGAGAAATTTTTGATTTGGTAAATGTTTGAATGTAAAGTTATTAATGTGCTATAAGGTAGCATTAATTTTGGCACATCC

CCAAATCAGAAAATGTCTTAAATAATTAGAGACAGTGATCTTTTGTCAATGTTATGTATATTTTTTACTAAAGAGTGAGG

AAAAAAAAAAAAAAAAAAAA

>Dorsal FJ998202

TGTGATTGTGTGTGTACCAGGAAGTGTGACTGCTAACTGTTTAAATGAACTGTGTTCTTGAACACAGAATGCGATATGGG

AACAAAAGTCTGCATTATTTAATGGAATTAGTGAGAGGGATGTTGTATATGTGAAATATTTTTTGGTGTCTCAGATTTAA

AAGCGGCATTTTTTGTGGGAACATTTCATATTTGTGGAATTAGAAATAAGATTATGTTTTAGCACCAGTAGCATTTTTTA

CATTTTGGTATATACTTGTGCTCACAGTGTTCCGATTCATAAAGCATTTAATCAGATAATGATTTTATATTCAGATTTTA

GTAGTAAAAAGTTTATATTAAGTTTAGGCAAAATAGTTAAATGCATTTTGTAGTTCTTGTCATTATTTAGTTTAATGAAA

ATCTTATTGCTAATTGATAAATTATAATTAATGATGATTTCAAGAATTATAATTAGAGGGCAGATTGCATGTACTGTTAG

CCTGTTCAGTTTCATTTCCACTGCACAGACTCACCTTTGGTCCCTGACTCTAGTATAGTTGGTCAGGCTCTCATATTAGG

CCAGTGCTGCCAGGGATTGCATGAATGTACACATCGTACCCACTTTGAGTTTACTTTTTGATTGTCTTTACATGTAAATG

TCTCCACAAGTGCTAAGTTGCCAAGGAATCAATTACTGGAAATATCTTTCTCACCTGATTACTATTCTCCTTGAGTTTTG

GAAGTATGCTGTTTTATCTTGTATTATTTATTAGTAATTTCAGTAGCATGATAAGTATAAAATCTATGATGAAAATGACA

GCATTGATATTAATAGCATTGCTAAAAAAAAAAAAAAAAAAAAAAAAAAAAAA

>Relish EF432734

GAAGTGACATACATTTCATTAACCTGAAATGTGCATATCATAAGTGCAGAACAATTTTGGTTATTGAGTTCTTACTGTTA

GATTTTCATTCTTACCATGTACAAACTTAAACTTTTATATGGGGGTTCACATATTGGGAAGTTTACTTTAGTTTTATTTG

TTTTCACCTTTTTTTATTATTTTACTATTTTCTTGCCTAAAATATACACTAGATGTATGTTAGCTTTGGCTAAGATGCTG

CAATACTACTTGGATTGTATTTCATAGGTAGGTGAATTGTACAAGTATATTTGTATTGAGATTGTTTTATGAAAAAAATG

TTCAGGATTAGAAAAAAAAAAAAAAAAAAAAAAAAAAAA

>sRelish FJ416145

gaaccttgtcttacactactgttgtattttcattccaattaaatcttgggaagcatttatatatgtaatctcatttgtga

ataaaaatattcaataacaaaaaaaaaaaaaaaaaaaaaaaaaaaaaaa

>IMD FJ592176

GGACAGCTAATCAAGGAAATTGTAATGTTACGTTTTCTTTTTTATATACCTTAACTGCTTGTGTGTGGTTGTTTGTTTTT

TCTTCTTCTTCTTCGAAATAATACACTGCTAATTGTAAGGAGAGATAAAGAAAATAGCACAGGAAAGGAAATGGTCTGGA

ATATGTTGTACGACTTGCCGGGTTTTGTAATGTGTCAAATATTTCATAACGTCAGTAAATAAATATTTTCATCTGCAAAA

AAAAAAAAAAAAAAAAAAAAAAAA

>IKKbeta JN180642

TCTTCTGCTGAAATATTATTGATATGAAAAATTTAATATGTTTACTTTACATTCTGTAGGTATCTCAGTTACTGAACCTG

CTCACGGACTAGAAAACTGATAATAATACTTTTTACAAGTGACAGGTTAGGAGTACTTACTTAATATAAAGTAGAAAAAA

TTGTGTCCTGTATGGAGATATATCACCATAAATATTGTACCATATAATTTATTTACAATATATTCAACATAAACAGTTTA

TCAATTACATAATTAGATTGTTATTGGAAATATCATAGTAGATATCTATATGCAGCAGGAACTGCTTTGTTATGTCATTT

GGACACCTGTGGAAACTAAGAGGCACACACAAACAGATACTTGCACACACATACACACACATATGCAAGTGATCAGATAG

CTATTTTACTTAAGAATAAAAAATAGTGACTTTTTTATGATGGTTAATTTTGACTTGCTAATGTGTGATATGTGCTGTAT

ATCTTATATTTTGGGCAGTTTTTTTCTTTTTCTTTTACTTTTTCTTTTTTTTTTCCTCATGCATTCATTATTGCTGTAGA

AATATCTCACACAAGTTTGGGAATTGCTAGGCATTCAAAGGTATATGACTGAACTTAGTGTCTCTGATTTGCATTTCTTG

CTGGCCTGGAATTAAATTTAGCAATTCTTTAATTAAGGAATGTTTCTTTTCCATACAGGAGAATACAGGCCAATTTAATC

ATTTATGAATGTAAATTATAAAATTAATAATGGAATGATAGTGCTTTACATTGCTGGTGAAGGTATAGTTTGATTGATCA

CTAATAGCTTCCAGCTTTTATTCTCTAACCCATAGATCAACTTACCCACTGAATCTTTAGTAAAACAGTGAAAAAGCAAT

TGTAAATATACAGTTCAACTTATTATTTTTTGCCACAAAGGTGATATTTAAATACATGTGACATACATTATATGCATTTC

TTTGTATTAAAATTACAGAGATTTTCTGCACATCAGCATAGCTTTATGAGAGATATGGTCGCTTTGTTAAAATTGTCCGA

GCCCTGACCTAATGAACATTCATAACACTGACATGCATATTCACAGAAATAAATGCATATCTACTGTATAGGTAGATATA

TATAAATGTGTATATACATATGATAGATAAACATGGGTATTTATTTCTGTGTATATGCATGTTTGTGTATACAAATATTC

ATTGAGTTGGGTTTCGCACAATTTTCACAAGCCAGCCGATAATTTAGTGACTGACAGTGTTTAATCAATTTTTTTTTTCT

ATTTTGAACTCAAAAGTTGCATTTGCATTGATCTGAGAATCTATATGTCACATAAACAGCATAAAGTGCCTTTCCAAAAG

TATTTATTTAGGAAATTAGTTTATAAAGAAATATGAGAGAAAATCTTCAATGAAAGTTACAGTTAGAAAGAAGCTCAATT

CTTGTGTTCTATTTTATTTATTTCATTATCTACAAAGGTGCATGATATTAGGTGCCAATTTGTTTCTCCAGCTTGTATTT

CCTCTTTTTTTCAGCCAATTTGTGCATGTATGTCTGGGTGCTTGTTTGTGTGTATGAGTGCATCATTGTATTTGTATGTG

TTGCTATTGATGTGGGTGTGTAGAAAGAGGTATGAATGAGAATAAAGATCCTCATAGAGCAAGAAATGTATTTGATTGGT

TTCGACTATATCTTCATTAGAAATACAGGTATTTCTGACAAACATACGGTATTGTCAAAACCATTCAGATACATCTTGTG

CTGTGTAGATATTAATTCTCATTCATAAATTCTTTTGCATTTACCACAGTGAACACTGTTAATCATGTGGATGGGTGTAC

ATAAGCCTGTACATGTTTTTGTGTGCAATTACATGTATGTACGTGTGCATATGTGTGTTTTTGACAGACGTAAATGAAAT

GACTTCATTACCTGGTCACTCTATTCTTAAAACAGTTTTGTACTGATATGTTTTGAATGTTTCTTTTGTCACAAATGATC

TGTAATGCAATTATACATTGCTATATTATATTTACAAGTTTATATTTCAGTTTCATTGGTATATGTGTTGCAAAAGGACA

GATACTTATGTTATTTTTAAATTGTTAGTTTTTTAATACAATAATAACTTGCTGATATTATTTTGTGGATAGTTAATAAT

GACCATTTTTGTTTGGTGTCTGTGATACCAAGTGTTCAGTATACAGTATAGTAACATAAACAGGTGCTTGTGCAAGAACT

AGTCTTGAATAAGATGTAGGAGTGTGAATTCATAACTTTTTCTTAATCACAAATTAGCACTTTCACATGCACACACCCAA

GATGAATAGAGAAAAATGTGTCATTATATAATAATTTTTAGCACATATGGCTTGAAAAGTAAGTATCTTGAAATTTTGCC

ATATTTTTTTCACTGTTAAAAGATTTGAATAATTTTATTTGTGTGATGAAAAAAATGAATTCATAGATTTTATTAATGTA

CTTTGTACATGTAAATATAGATCCATGTAGATGAATTGCGACTTGTTTACTTGAATATGCTGCATACAATATCAAGAAAT

TTTGGAAGGGATATGATTAAAGTCCTTTTCATAAATTCTCTATATACTGTATATGTTTATGCACAGGCTTCTGATAAGTC

TTTCCTCTTTGAACTGGGTAGGATATTGTTGCCAGTGCCTTTGCTTGCTTTCTTTTTTTTTTGTTTTGTTTTTT

>NRF KY864366

TAACAGGGGGGGCAGTTTTATGTCAAAAGGAACACATTGCAGGACAGTGCTGTTTGCAAGGAGGGCATTGTTGCTGAAGT

TCCCCCCCCCCCCATTCCCTTTTCCAAACACACTTATAGTATGCTATATATATGTATAAATATTCATGTTTTAATATGTA

TATTTATTTACACATATTTGTGTTTTCTTGAATTTGCTAGATTTTCTTTTGGTAGGGATCTTATGTTTTGACTCGGTATT

CCACTGCAGCCCAAGTCCCTTGACATGCAAGTCAAAAAGGGCATTATTACATAAAGTATATTTCTGTAGTGCTGTATTTC

TAACCATGTTGAAGAAACGAATATTTTAACAGTGTAATGCAGCTGTAGCGGACTTCAGTGTCATTATTTCATCAGATGTC

TACTTAGGAGAACTTTTCTTATCTGTTTTTGTTTGTTGTTCAGCTAATCATTTGTCCTATGGTTTTTGATATCTTAAAGT

ATGTAAGGTATACATACATACAGTATAATGTTTAGATTAAACATTCATTGTAAACAAGATTTATATTACACAAATTTCAT

AATATTTTGTGTTTGAATGGCATAATAAGAACCCAGATTATTTAAGTGTTTGTGAAGTCATAGTATATTTTGATTTTTTT

GAGGCATGAATAGGTAAAGTAATCTTTATGTTATTTATTTATTTATTTTTT
