# Supplementary material for: miR-10c Facilitates White Spot Syndrome Virus Infection by Targeting Toll3 in Litopenaeus vannemei
Source: Front Immunol. 2021 Dec 7;12:733730. doi: 10.3389/fimmu.2021.733730 (PMC8688535; doi:10.3389/fimmu.2021.733730)
Supplement: Supplementary File 3 — Hox homologs identified in L. vannamei. [file DataSheet_3.docx]

>Lab Genbank accession NO.: KM496275

tgtgtgtgtgagtgtgtgtgtgcgaggtgagccacggccccatgaacaacaccaacatggtgtacggggtgtgtaacgcc

gagtcaggctatcatagtcaggcatactcttccgaattacaacactactatgcgcagtgtcagtctcacgaggttgtaca

gggccagacctatatcccgcccgccctcacgcccacggaccaccaccgcccgcagcacacgaacctgcaggacgcccacg

tgccctcgtcgtacaccaacttggacgattacagcagcgtcggataccccgggcacgcagcaggaggcatgagcgccgca

cagcaccacgtcacgcagggctacgactacacgagcgcggtgcaggcggcggccgtggcggggggcgtcggcgggcagac

gggcgtgatgtcgccagcaagtgcgggctacccgggctacttggaccccgccgcccagtaccgacacatgagcatggggc

tctacgcccatcacgagggcgtgcgagaggcgtgcggcgtgggcggcatgatgggcggctcgccacacggcctgccgcac

caccagccgcagcagacggtccccacctacaagtggatgcaagtgaagaggaacgtcccgaagccagcgcccaagacgga

ctacggcggcttcggcggcgggacgggacgcaccaacttcaccaccaagcagctgacggagctcgagaaggaattccact

tcaacaagtacctgacgcgcgcgcggcgcatcgagatcgcgtcggcgcttcagctcaacgagacgcaggtcaagatctgg

ttccagaaccgccgcgccaaggacaagcgcctcaaggaggccgagctggagcgccagaaccgccctctgtacccgaccta

cggcggcctcctccccatgggcggcctcgtgccctcgctggccctccagaggcccctggtgcccctcctcgggtagagcc

cctggggagacgcccttgcaagtacctgcagcagcccaagacagccccggcgcccctgaggcctcctcctctccgtcggc

cctcctagtcacccgccgccacgggtctgcaccagctgccaccccctctctccccgtcggtgactctcgactgcacgctc

tgtcacgcccaccgcgcatgacaggaatgttgttcacgcccacaaacctcccacatccacgcccattatggatatctcag

tggccgcccgctggaagacggagatatagactgtgataaaaaaaaaaaaaatgttaatagataaatacttttttgtaaac

atattttttaaaaatgttggatattcttgtgaatttatatatatacagtattcgtaataaaagatgaagatgaattttat

atcatgtttctaat

>Pb KM496276

gccggcgggatgaagggtggccatggtggcggtgggcgtgggtagtgggcggcatggagtcttgtgtgtcgcagcactac

gttaccccgggcgccacctccctgcccatgggcgaatacgtccaggtgcctcccgacatgggcggccacccgtgcacccc

cgcgatgagctcgtacctggagtcgctgcccccgggcgtgggcatgccggagtacccttggatgaaggagaagaagacgg

tgaggaagccagcccagcaagggcgatacctgcaaggtatgcccactccacccggagccacgatcaactgccctaccgtt

gccacagagcatcctatcaaatcttatgtgtcaccaggtgtccccgcccaacggcaatactccaacagatccacggagaa

cggcctccctcggcgcctccgcacggcctacaccaacacccagctcttggaactggagaaggaattccacttcaataagt

acttgtgtcgaccgcggaggatagagatcgcggcctcacttgacctgacggaaagacaggtgaaggtgtggttccagaac

cgccgcatgaagcacaagcggcaggccatgaccaagagcgacgaccccgacaagaaggcgggcggcggccgccaccgcgc

cgacgacgccaccaacaacaac

>Hox3 KM496277

gcgccttcaagcgcctcgtttcgtgcttgtgggattgcttgcctcggggcgctcctcggggtcctcggcgaaggagcggg

cgaaggcaattcgagggcggaaaaatcggggcgtcgtcggccctctggcaacgcgcggtggcaaggggcatgagtgccag

cccgtcaccctatcccgcttgaggatcgtgccagagccagcccgtcgctgccacctccatccccaggggacccgagtgcc

cgtcatgtgcccgtgacagcgtgccacagacatgagtggcaccctcgcctctctcacgcactgtcacgccgcttcctgtg

acctcaagttcaattaaacgaatcggatttcccctcaaaattaccaacaaatcccagtgcttttatcctaataaaaagaa

caacaactatccactgaaaaggaaaaaaaaggaaatattaacttctcagtaaattacaagaaatcaagagcaaagacccg

ggcaggactggcatcctcctgcgccgcgcctccgcccatccctcatgcccgtggcaggagaagacgtcccgcacagcctc

agctctcccgaagtgccaagcggtgccttgcgccttctacaagatttattgctcggccgggcctggggggccaagattta

tgtttgcacgccggcactggttgaaagaagctgttgatcatcccaataatctttaggcgagcggctgacctcgttcctgg

cctgtgattggctgagcctgggcacgtgacctgaggtcgagaagatgggcgagccgggccaggggtcagtacacctggtg

ccgtgtgagacgcaggacataccagcggccagcgcaggcgtcgtcccccctcccgcctccgccatgcagaagaccttcta

cgagacctacccggcccagccgccggcctacacctcctactacgagaacgccttcaacaacggctacggctacgacatgg

ccggcgccgcctgcggacagtattacgacgagtacgtggactaccgcgcggcgtgcggcctcgcctccaacatgatgccc

cagcacatggtgatgccccagggccaccaccagccggagtacggggcctacatggccggcccgcagatgggctactgcga

gccctcgcccgtgggcttcaacaaggagtacatggcgtgggcgagggaggcgcagcagcagccgcgcccccagccgggca

agaagagccccccgcccgtgtcggtcgcagccgcccagcccccgcccgtgcagacgcaggtgcccgcactcccgcagccc

ccacagccgcaggcgcagcaccagccccctcaacagccgcagcagcagcccccgcccttgcagcacaacacgggcgtgca

gggtcagccgccgcaggtcgtcacgcccccgaatcaactcacgcccatctcgcttccgtctggcacagagtacgacggca

gcgacggcgtcagcgtgaacggcagctccggagggcagggcggcgggccggccaagcgcgcccgcacggcctacacgtcg

gcgcagctggtggagctggagaaggagttccacttcaaccgctacctgtgccgcccccggaggatcgagatggccgccct

cctcaacctgtccgagcgccagatcaagatctggttccagaaccgcc

>Dfd KM496278

gcgttggagagatacggacggcggtcggcggacgggccgtgcacagtcggccagcgatagtttaacaacacacacattca

cacacgtacacgagccggagcggcaactcggcgaactcgaggacgtcctagagtgtcctttcctcggcgagcctcggagt

cgtcctcgcgactgccaccagttgcgccgaagaggcacaagaagcgcggcggccgagggcggccttcgcggccaaattgg

tccaagtgttccccgcgtcaacagaagagcaagtgtggccaatggcggctgcaggcagacctagtaagcccacttgagtg

gcggatcctggagaggggtggggggccaatcaccgaccgcgcacacacacacacactcgtcttaagtaagaagaggaggg

gggcatgttaacccttgcctggaggtgcgcagtggcagtgcgcaggtcacacacacacatggggcccgggcggcagtaaa

tcaacgccaatcatcgtctggctgttggacgaagcacacgtgaacggagcccatcgatcagtgcggagcgtgaggcttgt

gagtgtgggtgtaagggcaggtggtgtggtgcccctcagccgtgccccctagtgcccgcccccgggtcctacagctgctg

ctgccccgtcgtcaggagaactccacccctggctcagtgcttatcggagacttgtggcgatcggtagggtggtcgggtcg

ggacgcgcgcagagcccgaccagccctggtggttcgttctccccattcattaatcatggtgacgtagccagcgagggtca

cagtgagctattcgcattggtgctcgcaggtcacgtggtatgctaattgtcggtgcaaggaggagtcaaagccaacggtc

gaggcactactcacaagtaatattatttgggtccaattcaccaaaaattaatgacgatgagttcgtttttgatgaactcg

ggtccgtacgtggacccaaagttccccccgccggaggagtacagccagaactcttacatcccgccgcagagtgactacta

caacgctgcccagcactacccctaccatggcatgcaccaagcccctggcatgcagtatgggcgcgatgccatgcagtaca

accatgcagggtattaccagcagacgtgcgtcatgccccagcaccagcccatggccgcccacatgagcccgcaggtcgcg

ccctgccacagccccctccagcaacaccaagtgcccccgcgctcgcccgtcgcgtccccggacccctccgcgggcggcgc

gggcggcatgggcgctcaaatgggccagcacatgggcggggcgggcgtggacggatcccccgaagaaaccgtgacagagc

tcgacgcgaacggacagcctgtcatatacccttggatgaagaagatccacgtcgcaggtgcagaaggggcggggacccta

tttgccaacgggtcgttccagccggggtgcgagcccaagcggcagcggacggcctacacgcggcaccagatcctggagct

ggagaaggagttccacttcaaccgctacctgacgcgccggcggaggatagagatcgcccactcgctgtgcctgtcggagc

gccagatcaagatctggttccagaaccgccgcatgaagtggaagaaggacaacaagctgcccaacaccaagaacgtgcgg

agaaagaccaacccatggccaccttgcgcccttaaatgaaacaaaaacgaaaggaataaactgtcacttcgttccttcct

gaaagacttggtttatttagggacattttcagaggtgatgattctgaaggaatctgctttcggagagatatagcaatagt

atgtttttttttttctctcgagtatatcgtgcgtatagtgttccaaagaaaatactaaaaaaatataaaaatagaaaaaa

aaggatcaataaaataattcacaccttaaatctcagtgtgtttatcccagagtctttataaggtggcgtctatgaaacaa

ggaaacaaacaaacaaacactccataaaaaaaaaaaaaaaaaaaa

>Scr KM496279

ggcggcgtcccctcctgctagcgcaccgcccgcacgccgtctggtcgccacacgccgctaactccccgcgctgctcacag

atgtcgcggccgcgccggccggctcgctgctgcgcatccccggccctcatgctcttatttacggtttgtttatagtcgtg

catcgtcacatgggcgctcggtggggccaagcgcgctccgactgctcgtaatttatccggacggcgacggtggtcggacg

ctcggagtgggtgcagggctcgcgcagggtccgcgggtcgggccagcctcgttgcggccgcttccactctcattatcttg

ttaacctccactcatgcgcccattcctattggttgctgtcggtcacgtgttagtattccgagcccatatatggagtaatg

tgcaagtgctagagaggtttcccttcgactagtgtcgccataatccatcaacattgggtggaacagtggacagaactgca

ctcgtggagcaaatcacttgcggacttacgcgatgagctcctatcagttcgtgaactctatgtccgcctgctacgggcag

cgcggacaggacgccagtggtgtgaacacagactactacggagcctccgtcaatagttacaacaactgctactcgcctcc

actacagcagtacggaggttacacaccgtcgggactctcggctgtcccgaacggcagcgagttccctggaggaggcagtg

cgggcgtggtagtgacttcggcgtcggcgggaggatcgtcagtgggcactccgagcggctctggcggcaacaccacgccc

gggccctccgtgcaggggcgcctccaccagacctccacctcgcctgccgccacgccgcaggcctcctcctccgcctcctg

caaattcgcccccacgccagagtcgtcgacggcgaacccggtcggctccccgcaggacctgtccgtctccgccggcggca

acgggccgggctcgagcagctccggaagcggctccgagcagaacagctctagtcagggcggcgggggcggcagcagcaac

tccggcggaggaggcgaaggagccgaaggaggagagagtctggagggcgcctctggaagcgcgggaggctcgtcctccgg

gaagcccgcccctcagatctacccgtggatgaagagagtgcacctcggccagaatcagaaggatggcggaaaaggtacag

tgaactccaacggcgagacgaagcgccagagaacgtcctacacgcggtaccagaccctcgagctggagaaggagttccac

ttcaaccggtacctgacgcgacgacgaaggatagagatcgcccacgccctctgcctcaccgaacgacagatcaaaatctg

gttccagaaccgaaggatgaagtggaagaaggagcacaagatggcgagcatgaacgcggggatgggcatgcacccacagg

cgtaccaacatatgcaccaccaaatgatgcacccccaccaccttcaccctcatctagcggacttagaaacgaaaggctac

tactaggggtctgcctggtcgtggtaactcatatgcagacgccgtagccagcgagagtgggtggctacttcggtcggtat

gagagactgcgactagggtgtaccggtggactccttccattcgtcactcacggcctggacttggactacggactacggga

ctactccttgtgaactttcgaagcaaaactattgtgtttgtttttttatatatatatatataacattggccttttaacag

aagaaaacaggaa

>Ftz KM496280

ggcgtgggacagtaggcctatcagtacggccatcatctgtcgcccgcgcgctgacatgagctcctatttcgccaacgtgc

cctccccggcggccaactggggggcgccctcgcaggaccagtatgcgtacatgcagagtgctaaatacccctgttattcg

gaccccgcacaggcctacgcgcagtatggcggcatgcaaggctaccgataccctagtgcatacagctgcgccctcgggaa

tatggccgccaccaagggcgcctacgagcagagtcccagccccgccatcaccgccacggccgcgacgagtcccctggggt

acgaccagggccataactactacatgaacagtgccaccgccgccaggaacgacgaggcggtagcgcaggccatgagccaa

gcagtgccagactccctcagcccagtgaacagtgattacagcagtgtcaagatgaacagctacgtcgccgcgaaccccat

ggcaggcctgacgcccacgcccgtgagttcagtgcccgcccatcccctcagccccagcgacgccctcagccacgcctatg

ccacggctcctgacgcggtgatgcagccctacacgtccaaaacacagtccccggccaactactaccagtgggtgaaggcc

tatcctgcagcagggcaagaacccggttcgggccccaagagaacccggcagacctacaccagataccagaccttagaact

cgagaaggaattccactacaaccggtacctgacgcggcggcggaggatcgagatctctcacgccctgggcctcacggagc

gccagatcaagatatggtttcagaaccggaggatgaaggccaagaaggagtcgaagatctcttctgcaggcggcgaaggg

ggcgctgccggcgggggaggaggggaagagagcgaggagaaggaagtgtcgagtgatcctagttcctcgccggagctcct

gacggcgtcgctggcggggtcgctgacggggacgctgacggggtcgctgacggcggagctgacggcgacgtgaatggatg

agcgaagcccggaggagcacagcgcgtcaaacgggaggcgaagaagaggggaaagaaaagaggggagagggaa

>Antp KM496281

gggggcactgcggccaggagtacgacccccgcatgccccccacacacccgtactacactcagcaaggctacccgcgctac

ccgccctacgacagactcatgaacaattactacaacgcgcaaacacctcagcacccacagactccacacggcatgcaacc

gcacgacgcccacgactaccgcgacccctcgcccgcggcgcccacgtgcatgggccagcaagcgtccccgcccgtgcagc

agtactcctcctgcaagatggcggggcagcagcagggtccgccgcagcaaccacagcagcagccgggcatggatccgtcc

ggaggtcctcctcaagacacagcccaccacatggccgcgcaggaacatggccagggatggccagcccagcagccgcagca

acaaaatacccaagccgccctcccttcgccgctctacccgtggatgagaagtcagttcgctgagaggaagcgtggacgac

agacctacacgcggtaccagaccctcgagctggagaaggagttccacttcaaccggtacctgacgcgacgacgaaggata

gagatcgcccacgccctctgcctcaccgaacgacagatcaaaatctggttccagaaccgccggatgaagtggaagaagga

gaacaaaaccaaaggagtggagaacggcaactccctctcagagacccccacccccacctcccccacccagtgacctccga

gctgcgggtcggttgccaaatattgacctagggtgtgcaaaagtgccagtgcagagtgtgtgacccattttatactggat

aattaagtgctattttacatagtctacgtgtatgtgctccgtatgtacagtgcatctccaaaaaagaaaaaaaaatattg

gcaagtgttttgaatataccagtgactacatgtataagccaggatgacgtcagcgaaggcgccatagtgtaaagtgtatt

ctgtgtattatcggtgttcaagccgggaaacagcctaccagtgccagtaccacataggggcttcgggagccgggtgaagc

agtgacgtggcagtctcctattggtgtaagctttatgcattagcagtaacgtaaagaaaaaagaaaaaaaaaagtcataa

aaaaagagttcccaaagctacgttgccacctagcgacaatacgtcaacagcgaagactacaatcattccaagaaccagat

gagtagccgttttctgtcatctgtcatgtcatccatcacttgacaagctaccttgacccccaaccccccgagtccccaac

aaccatgtcaggcgccagtcccccccctcgctaaccaaaactaaaaccacacaggagagtctagctgtttgaacctgatc

taccgtttccagttgcttctcctactctggtattgtataaaataagagtttcgatacaaaattgatgtttcccactagga

aaaacccagtatatataatatatgctagattgtagcgggaggcgaaactaaactaggtgttattgagtactactagtata

ccacacaggttacaaaggataatcagaagtgatcagcagcaatggtatcttccgaagtgatctctgtacacagatggacc

agctatgtagatggttctgtccaaaagatggttcgacaatagagatggtttccccccaaggagagagggctccctttcgt

atatagttgagccatctttgtttattttccttttttttaaatcaatgatcaagtcctccttgatgcaactggtccttcaa

atgatcattctagctatgaggaagggtcttcgaggtccacccactgatcgttccctttgacgtccaatacacgcccctgg

gggacagaagcgttcagccagtgtacccgtttggagtcttttcccccttcctcatgcaaagatggttccatgttcgcaga

tggtccgcttacagagatggtccctcaaaccccaccccccttaccagatggtcgctctccggaaggaatagttgtatggg

caaaaatataagaaagaaaaaaattaagaaaacacgcgagaaaaaaagtatgaatagataacaacctgaaaccagtccat

atcacaaagtatccacatcttatgctcaataaatacttcgtaaattttctctctctcttttatatgtatataatttattt

tacttagatttttttttcttctttttggtttgatttccattttctatttatttacgttctgaagtcatatattttctcta

tcacttttttcgggaataaactgatttcaactgggggtacgtgcgttgatccgccaccataaaacaaacgaaatgttgcc

catataacgatattctgctgaacgtgtgttgcaagttgaaagacgggtaaatggaacaagaagacgacgataataagcaa

actaaaaaataacaaaaaaacacacaaaaatcaaataaaaaataatgtaaacaaaggctcatctatggaaggttttgtgg

gagaaagatcttggcttgccttcttggagtccctgtgcttgcattcgtgggagtcgaacgggaccaaagcagtcaaaatg

ataataaaacaaaaggttgtgtaagaagaataagaaagaaaaagaaaaagtggatctccatctcactgattttagatttc

attattcttttatcatttcattatctctcgcattcgttgtggacgtgatgagtagtcctttaagtcaatctttgaaatgt

atccatatttataaaacgtcatgctcatccgccatattcatctaccatctactctaccttcctccctctcctctacctcc

ccccaccctccccctcttacccgtctccgcctccatccctcttttctttctccttctctttcgtcctcttttctttcacc

tcctgtttcttccgagttcttgtcctctccccgcttcttgtttcttcatcattctcttcttccttctcctcctctacttc

caaaaccactccatcttcctcctccttttccttttctattattaagtttcttcccttccccttctttctccaccaccaca

gccacatctttttcttcttcctcttcctcttcctctccctcctcttcttctccttcttcctcctcttc

>Ubx KM406487

ggccagtgtgcgccagccagccagccaccaccgataatcgcgcgtgtgtgcgagtcggccggtcgtgacgtcactattac

ggagctagttcttcccgcggactcccgtcccactcacagtcgtcctgcggccgcgtgacgcaacccatcctcattggctg

cttctgcctctccgggccgtccttcatatgtttacacatagcgactgctatgagcaaatttaatccagcacatggtgtgg

tagcgcgggtaaaatttacaatttgaaaatcggttggtgtgttggtggtgatcacggtgtgttactggaacttcagttgg

ttcttggtgttggtggagtgagtgtgtcagtgggtgcgtgtagtgtgttaccttcataaccacaaccatgaactcctact

tcgaacagggcgggttttacggcggcggctccggcggggaccaggcttaccgcttccccttgggcctcagcgtgagcccg

tacgggcagccaggacctcgtcaggatggctacgacaccacggccgcctcctgcaagctgtacgcgccccctcaggagca

catgacgcccaaccccttcaaggtggactgcgtcaaggatcagaatggctatggagccaccaaggacatgtccgggggct

gggggcccgcggtgcgccccgcctgcaccccggacccggtggcagctcgcggctaccctccggaccccagcacgtccccc

cgggaccgggcagcacacgtgggcgggtggaacacgtgcgggatgacgcccgtgtcccagccccatcagcaatcccagca

gccgcctcagcagaccaacaaccagatgaaccaggcgccctccaacaccaccttctacccctggatggccattgcaggag

ccaacgggctgcggcgccggggacgtcagacctacacgcggtaccagaccctcgagctggaaaaggagttccacacaaac

cattacttaactcgccggagaagaatagagatggcgcacgccctgtgcctgaccgagcgacagatcaagatttggttcca

gaaccggaggatgaagctgaagaaggagatccaggcgattaaggagcttaacgagcaggagaagcaagcgcagagccaga

agatggctcagcaacaacagagcgccagcaatgcccagtccatgacctccagccagggagcgggagggggggctggcgcg

gaccagaaccctccaaaccccacgcaaaactaacgacacaatttcatctataagctagttttaaggaaactttgccgcat

gcggacgtgaggacggaacaaagactgcaaagctaccatcggcggagtcttttccgcaggccgccggcgccgccgccgcc

gctaagagcgcctctgacgtcacgggacctgcggtggccacgccatttcccagaccgcaaattccgcagtttgggacttg

cgtgatccaaccctaagcctaaacccaccagagactcctggtatacccacctgcacacccgatactccgcgaaatcctcc

gaaaaagcgggttctctcgtgctgtgcggctcggcggcgaggaggatctcacgacagtgctctgcagggaaggacgaggg

acgagcgtttcaggaggagccgcagataggccgtcactggaccgaatatcggggccgcagttctatgttcaaattaccaa

tgacaactcacaatggtcggcctgaaatgtgggggctccagtgaggctctctgaaggcaggaagtataccatttgctttt

cgggcaaacaaattcatcaggggttctgggttaccctgcaggagttactgtcatattcacattgtatcctggtaatttta

gccatgcctgtgtatacgtatttgctataagtgtttgtgttatctctaaataacggtttttcttgtctgtgtaatagcct

agacgtaaaggataaagtaaaaatgaagaaagctctccagcttttctaaatgtatgtgtccgtgtatttaagaaaagtaa

aaagtttttttttcagaaaattaaaaaacgaaaaaaatataaaaataaaaaatatataaggttctggagagattttacaa

gctaaatcctaagtcagtcagatctgtcgtttcagtactaccaatgatttgtaggaaaacaatgatttgtaagctgatgt

taaaacagttctttagggaagaaatccactgaccatttagcagttgagatcataatatttatgtagtagcgttaaataaa

aatatcaatcatgaaaaaaaaacaaacaaacaaacaatgctgaagtgtaaaaagagaaaacagagataaaaataaaatat

aaggatccacgcacaagcctctacttcgtgttagaaggggtatcgcagtcctcctgaacttaagagtcgggcgcgaggtc

ttgttggcctctggacgccggaatcggagccagaaccatgaacagctttgaaatgggtggcttaacttttgatatggttg

aaatcgacgtgaatatgaagatgaaaaagaagaaatattcttatagtgaaacatttttaccgcctgtgctgccggagaaa

tattatgtgcgggtcgagtagataggtggtgcgtggatgtatgtatatgtgtacatgtacgtgtatgctcaagcagttag

ggccacccagacacacgcacttacgtggaacggaactgatagtttctatgttaatacagtaatatgatcctgtacaaatc

tacagtaatgttgtatagtcagcaagtgtccgtcggaatctgtcacgtcaagacattcccgtctggtcgtgtcaactgtc

atgaggttttgatgaaccaggttttcattcatgggtgagcccttgggcgaagaagatagagttctgtcttatttttgtcc

gatttaat

>Abda KM496285

accaaggcgagcgcttattggttcgctgagctcccttacctactgattgggcagacttgtttaccgctcgcggttctcgt

gcctggtgtctaattcagaaaaggattcatttgccatcacaggtagtcgaggctggtgtgtacacagttgttactaagta

gttaccactgggaagaattatgtgaacaatgagttcaaattatattgatagtattctgccaaagtaccaggcggactccg

ctgctaacctggtcaactacaattctcaagccagaagtatgtacccctacgtgagtgtcacttcacatcagctgtctgcc

aacgcgccctccaacatgtcgcccttcagcgctatgacggcgaccacagacgccgaaaagcagtgtcgatactcccagac

tggggccacggacatgtcccaatatggcctcaacctgcagaactgtgccaccacaagcaacatggcacagtacttccacc

agaacaacgccactaaccctctcaactcctgcagtcagcctacagctcccacacctcacatcccggacatcccgcggtac

ccatggatgtcaatcacagaaaaccaatggcgaggtctgacagccaattggaacggcctgccatggaactttggggcatt

gagaggtccaaacggctgtccacgacgacgcggccgacagacctacacacgcttccagaccttggaactcgagaaggaat

tccacttcaatcactacctaactcgccgacgaaggatagagatcgcccacgctctttgcttgaccgagagacaggtaaaa

atctggttccaaaaccggcgaatgaagctgaagaaggagctgcgggcggtgaaggagatcaacgagcaggtgcgccggga

gcgggaagagcaggagaagttaaagcagcagcaagacgacaagaagaccaacaaggaccaggcgtcggcgggcaacaccc

ccgccggagcagccaccaacgcttcctcctcgtcctcttccacctcctcagcgtcggcgggaggaggggcaggggatacc

aaagctgcgacttaacgttatggtgatttctccccagatgggcgtgcctatttaaacagaaatttccacttcacgcccac

acaatccaatgacatgcacgcccagcaactccctctcacgtccacctctccagaagatgttgcactctccctcaccccga

ccctcagcaccgccatggacctggagatgtataactcgatattaaactaagaatttaaagttttttttttagaaaataaa

agtgcaatattggtgatagaaatttttgatagtgatgattttaaagccaaaaaattgataagttctcgatgatgattgaa

agaaagaaggctggaaatgatgtcgaaggcggacggctgcgtcgagatttccgaatcaaactcgagcctcgtcctcgcag

cccgacgcctcgcgcctccgggttccagcctgctcctgaagctccaaaggcaaagccgtttgaggtgcggggtctggaag

tgacaaggaaacttctggagctcgacaactatgtgttcttcatttgtgaggcacacgtgtgctctttggacgacgaggcc

acacctccagcaactacttcttcaaatagtgaacaaaagaataaagattacagagacagcaattccaaaatataacaaat

agccatcaaattctgaatgcaaaagcaaagaaacaaagcagtaatatccagatagtctatcacacacatttgcttcggga

acttcctgcgtgtgacctttgacctcacaccggggccccgaggcaatgcatgacctaaggacatttctcctactcgtgaa

cttgacctctgaaagtaaaaccaagtaacttcatggttcatttatatatcaacaccacgcgggttacctttccaaaacgc

cctgtcgacttacaaggctcatggagatagtgaacgacagtaggattcctttcaaatgacacgccgcaagtgcccccggt

cgggtaaagtcctctgccacgccagcctgtcttgtaagtcgacagcgtgtatcataaaaaagtctctcgatttggttaga

cacttgagatggttcttttttatatattagatcgtttttctttctttagtctagtaacacgatcgttatatcatgaaaat

gacaatgataagtaaaatccagtcctagaattttcattagtcaccatcttgagtagctaagccaagaagagaggaagaag

ggaaacatatatgatactcattgcaatggatccacacggttcaagtgtcccggacgcgaaaatgactcttgatagcccat

ctagattgttataagttaatagtgttgttcatgacaggatgccagtgaacagataatagcagatgattagctaatcaatg

gttcctattcccgatgtttgtgaaaaaaggaaagagaaattgaaacttcctcatgatttttgtacatttcgtgttaatga

ttaatgttgatttcttatcggcttctttcgtgaaattagaagtttttttttaccaatgattccagctttaagttcaattt

aaaaaagaggaaaaaaggcgcagattggaactgtttgactgattggaatgcggtagttcagtgactgagaattgttacct

tgtaaatggattctatttatttgtattaattgttatgtttgacgtcggattatagggttgatcgccatgctggtgtacat

gcgtggtgtgcatggaaggtatacatgtaaaccttttttgttgtatgcatgttacccccaaagatgccatggctaagtac

aaagaaggagaatttgtatattgaaactctctgagcttcaattcgtattggagtctgtacactttgcttcctccttgcaa

aagaattatgaaaggggaacttcatgctattaagtctaaagtaaatacgattttaaagctttttttaagtcacttcagag

agtttcaactggaacgacaacgcatgtggaggcattagtgcaatagccggtatgctaaattagagctttaaagttgataa

atgtaatttctgggaaactgtggatgaataggctaagagaagaagctcgaggagtccaatttggtctcgaatacctctct

cctccgaattattttaactgtaatcatcagtgtgcataataactgtaccttaggccaaggagtgtgcataattatttgac

cgaaaggagtagcgaaacgttgtgagcttaggaaaggcaggcaagggatgactttgtgaaatctttttgctgactgtctt

cacgttttttttatgttgacctattaccttacgtcagtcatagggccggggtccttttgtatagtgtgtaagtagttgac

cttatttgccctagctcttttgtgatatcaaataaacaaaaaaatgaaataaaaaaagtaaattaacatgttcgagaaag

ccagggcaataactttgttcaaccaagttgatatgggataagatctcagatggttaaacagtggacataatttctttgta

gacgtgtgatcattgtcattgtttatgttttcactcttttgaaaatttatcctcatttaggctaaaatgttgagtatttt

ttgttttactttgtatatattcatagcgtctttgaggtatgtccatctgttatagaagtagttagtattgttatgggaaa

gtatgaaattatgttgataatttaagttattgagtggatgttgtcgtgaagtttgcctttctgtctgtcgttttcgtcac

aagataaagaccacaggatcacgctactacgcctcgactctcgattatgaatgtcacttttttcattcagttttcctttt

ctttgtggatagtgtaagatttctcaggtgttcgattcagatccggtgagccgtcagacggtgagactttgtgaaacatt

ctggtagataattaaagtgagaagagacagagagacaaacagaagaaggaagaagaacagggaagagcaatggttattgt

tattttttacttgtgagagaggtctgagaatccgaacggtaagaatgaaatcagaggtcggtgagatccattgcgggatc

ctgccacattcactatttttatgaactttatagggaattcaagcccattaaaacgggtgcatgtatccacttgatccact

ccatatccacagggtcagcatgacctcttgggggatcagaatacgtgttaacaataccagtgacgccgcctttgcgtcac

attgaatattcatcacaggcgacagtaaacagatatttggagacgttagagaaaccgtatgaaaagccgagtaaatctct

ctaagggataaaaggtaaaaggtgttttgtcgatgaaaatattgaggtttttgtttttttccaaaagaggaaaccgtatt

acaagttcacatcttttgttgttgtgctgactgctatttccagatgtattgcagtagtagtccggctcttctcaaaattg

catgaatatcgactaatttgaattgtgtattgttgtattcttaatgatgacttttcatggtaacaggtgatgcatctgaa

tgagttatacttttggatcatttgtagaaatactgacaacgggtgtattattttctgctaacaattatgtttgtgtcttc

aggtaaactaatgagtgttttcatgcacatagggttttctgttgctaataccattgcaattaccacagccctaatggatg

agttactccagatgaaacatatggttcgaaagctggtatgactggtgacaaaaaatacacacaacaaaatctaaaaatcg

actcggaaaacaaaaggaattaaagaaacaaattggaataaatgaaatttagaagaattggtatcttgaattcatgtcca

cgacttaccagagacttcaaagcgtgcaaatatccgactactgttgtttcagatccaacatttttttttctaagattgga

tacttctattccaaatggtacaatttctctttgagtgagtaggtggtaccaacacctcctactgactctaacgtgatact

gatatatgaacgactcatcttcctccttctcccccccccttctccctatctctcccctccccctctctcactggaaaccc

tccctctccccca

>Abdb KM496286

ctcgccgcggcctcaacacacgctggttgttttttgccgcggctactggtcccgtgccgagcgtggtggtgcacatgcgg

cggccgtgctagtgcacgccagctaatgtgtgtgtgttgttctctttttccgatcgtgtgtgactgtgcggcggcaccgt

ggcgactgtggactcactaccgtggcggcaccaccatgcagtgggcagcagcgagggaggaggactagacaggatgaacg

gagctttgtatgaggactcggccggcacacgaggcctggagcctacgggccaaacaccactacacatcccggccaagcgc

gtggccaccaccctggctgtgtccacgtacccgcacccgcccgagtgccccacggggcctgactctggcaccgcggcggg

tgtcatcaggcactcgcactccacacagccctggaactaccaaccggagcatgcagcagccacggcgcccttcgactccc

agtacggccaggccttcgggcgggacggggtcacgcccacgtactataacattgcggacaccagagtggctgaccgcaag

actctggccttctggcccaacaaatacgactacgcaccggggccatcctcgatgaccacggagtcatgccaagcctttgc

cgctcagacctggtgcaactaccctccctacgggcgtgtgggccatgtcgatgcccacggccagcccgtgccatacctaa

cggcggcagatggtgccccaaaatctgccatggaagctgccgcaggatatccccacgacggctacctgaggaactacccc

acagcagagactatgccccccgcaccttaccctccagcgcaccggccgcccttccccgtctcacatccaggcgcgctggg

ctccaacccgctggagtggacggggaacatgacggtccggaagaagcggaagccctactccaagttccagacgctggagt

tggagaaggagttcctgtacaacgcctacgtgtccaagcagaagcgctgggagctcgcccgcaacctcaacctcacggag

cgacaggttaaaatatggttccagaatcgtcgaatgaaaaacaagaaaaatagccaaagacaggcggcgcaggaggcggc

agcggctgccgcggctggcactccttcctccgggggcggcacccccggccaccagcccaacaccccccagactccaaaca

gcatcaagccttgacaagtgcagagttcctgtcacccctctgcccctagcctgcctcccgctcccccgcccctgggcgcc

cacgctgccatccccaccatctcgtcggatatgtgggcgtgaggtgctcaggagcgaccgcgggtggcgcgtagtgtcag

tgcccgtcatgactcggcatcactagacagtatgtggtgacgtcatttttgatgtgaccaggtttggcggcagtgtcgtg

gacaggaattatgaaaaatatatatatattactacgtcacacacaagaaagagacttgtccttgtgtaatgcaacccagt

gaaacttttttgagaattcaaaagaacaaaaaacaaaaacaaaagcagccctcacaaggcagtgtttgggcgtcgagcgc

ctggcccggctggcccggctggccacggcttcgaagagtcgacccactcgggccagaggtggtgtcgaccggtggtgttc

ctagtgactcagtgttgactctggccaagactagacgaagcgaggacggtgctcctcccgatttgtaaattactatttaa

cgtcaggtgtaataaatgtacataattccatttcgtacgctgtaggtattgtgcttaatgttccataattcatttctcag

tgtaccagagtttattgtgaaactgttactgcagaacccggcgttcctcagactcaaggaacagggtcgctttacccgtc

tcagattacgttgttatttcttgttatattttgttagaaagcgcagtgcaaggtcgatgtctttacgttagaaatagaag

catctatgtgagagctgaagaaagtggctcaagtgtttatcattattattattaataatatcatgattata

>Dll KM496284

gaccgaccgacgagcccgcgaaacgcccgaccaaccaaactggcgtcggcggaaacagccaccatgcccgaccaggacct

ggcctccaagtacatggacctgccgcagcaggggctggcgagcatggcccacacgccgccctacgcgcagcccttgggct

accagcaggcgcccaccccgggctacaacccgcccggctacggattcccgcccatgtacccgcagagctcctacccgggc

taccccatgagctcctacctcacgtcccagtgcccttcgccctccgtcgacgaaaaacccgaagacgaggcgacagtccg

tgttggcgggaaaggcaagaaaatgcgaaagccgcggaccatctattcatctcttcaactgcaacaactcaataaaattt

tccaaaggactcaatatctatcgctgccagagagagctgaacttgctgccaaacttggtctcacacaaacacaggtgaag

atctggttccagaaccgccgcatgaagtggaagaaggacaacaagctgcccaacaccaagaacgtgcggagaaagaccaa

ccccgcgggcgtcaccaccaccgtcacgcccaagaaccagcagcagaaccagaaccagacgcccacgacgcagagccagg

gcacgcccgagcagcagcacaaccagcagcagcagcaacagcagctggctgagttgggtcgcaaacagctggagcagatc

ctgcagcagctacaggagcagctgcaggtcaacctcatccagcagacgcagctcatgcagaccgcagacaagagcaaggc

gtccggccccttggcacagctgggcgcacagcagcagcagttggtgcagcagttacaggcggtgcagcggcagtacttgc

tgcagtcgggcctccatccgggtcttcatcaccacaatggagaaactcgttcaccccccggcagcggaagcgagggggga

ggcgcctggaaggagcggggcgagcgctccgaaaccccgtcaccgcaccaccacaacaacaacaacaacaataaca

>Msx KM496282

cgcccgcacgccctcgccagagatttctcatagcactcgtggattgttgttgcgtactccctcacgcccgctctgacgct

cgaaaaaaatggacaaatccaccgccgtcaccgaccagaagcccgctcagaagccgaagctgtccttcagcatcgacgcc

atcctcggcaacgacgacagcggcagcgcccgcacgccctcgccagatcccgagtgccgccccgcctcctcctgcagcgc

caccagcgacgtgtccttcgccagccacgactccgacaggtccgtgagcccttcggcggcgcccgcgggaaggcccgtcc

agagcaccctccccgcgcagtgggtcccggcgcacccgtacttccagttcgccgccgccgttcacggatccccgaccctc

cccaagctgcccagctccgtagttttgcgcaagcacaaggctaaccggaagccgcgcacgcccttcacctcggagcagct

cctgtcgctcgagaacaagtaccgcgagaagcagtacctgagcatcgccgagcgcgccgagttctccgcctcgctcagcc

tgacggagacgcaggtcaagatctggttccagaaccgccgcgccaaggacaagcgcctcaaggaggccgagctggagcgc

cagaaccgccctctgtacccgacctacggcggcctcctccccatgggcggcctcgtgccctcgctggccctccagaggcc

cctggtgcccctcctcgggtagagcccctggggagacgcccttgcaagtacctgcagcagcccaagacagccccggcgcc

cctgaggcctcctcctctccgtcggccctcctagtcacccgccgccacgggtctgcaccagctgccaccccctctctccc

cgtcggtgactctcgactgcacgctctgtcacgcccaccgcgcatgacaggaatgttgttcacgcccacaaacctcccac

atccacgcccattatggatatctcagtggccgcccgctggaagacggagatatagactgtgataaaaaaaaaaaaaatgt

taatagataaatacttttttgtaaacatattttttaaaaatgttggatattcttgtgaatttatatatatacagtattcg

taataaaagatgaaga

>Mnx KM496283

gacttaggtgatatcagaagcgaacccgacgtcgcaaggacagcacgtgtgctcgactggtgatagtgagaagatagtat

gtggacacgcttgtgatcgctcggacgtgacgcaccgcttgttgactgatgactcctcgatgagaaagcacttggacatg

tgcgtagcgattagagtgttcatgcataatttgtgaatataaacaagaaagttttataaacttcagaaaacactgaacat

agccagcggaggcgatcgcagccaagagtaggaggattctgtgccagggaccatgtcgaccagcgagagaaagtcctttt

gcatcgagtctctgctgtcccgtgaggctgtggttgcgagcggcgtcggcggcggcggccggggcggcaatatcagtccg

gcggatttaacctcgcacgaagtggggtcaccgccccacacgccgcccctgagccccctgccgtccccccactcctcccc

gctctcgtccgtgtctcccgccgtttcttcctcagcaattctaaaccgagcgactcttctcgggggggccactgcccccc

tcgtgccccctcacctttaccagtacccaggcatgggtatgccccctggtctgctccccgcccacgccttcacacccgca

gcatcgccacttctggacgcacaaaccttcaacgccctgaagtcgggggcggcgtccctgccccccgcggcgctggactg

gttcgcccgcgcgggactcatgtatcctcgcctccccccggaactggcaggcatgggtcagcacagcctcctggggaaga

cgagacgccctcgcaccgccttcacgtcgcagcagctcctcgaactggagaaacacttccgggagaacaaatacctgtcg

aggccgaagcggttcgaggtggccaccagtctgatgctgacggagacgcaggtcaagatctggttccagaaccggaggat

gaagtggaagcgcagcaagaaggccgtgtcggagaaccgcaaggagcagcgccagaaggacggcaaggagaactccggcg

ggaaggacgagcgcgccaaggaccaggccgggcccgggggcgcgacgggcacgcaggaggaggccatcgtcacgccggag

tccgaggacgagatcgacgtgcaggacgaccacgccggcagcgaggaggggggcgtggagatcaggggcggcgaggaggc

gcccctgtcgccccccagccccccggggtccctctctcagccccc
