# Supplementary figures and images for: miR-10c Facilitates White Spot Syndrome Virus Infection by Targeting Toll3 in Litopenaeus vannemei
Source: Front Immunol. 2021 Dec 7;12:733730. doi: 10.3389/fimmu.2021.733730 (PMC8688535; doi:10.3389/fimmu.2021.733730)

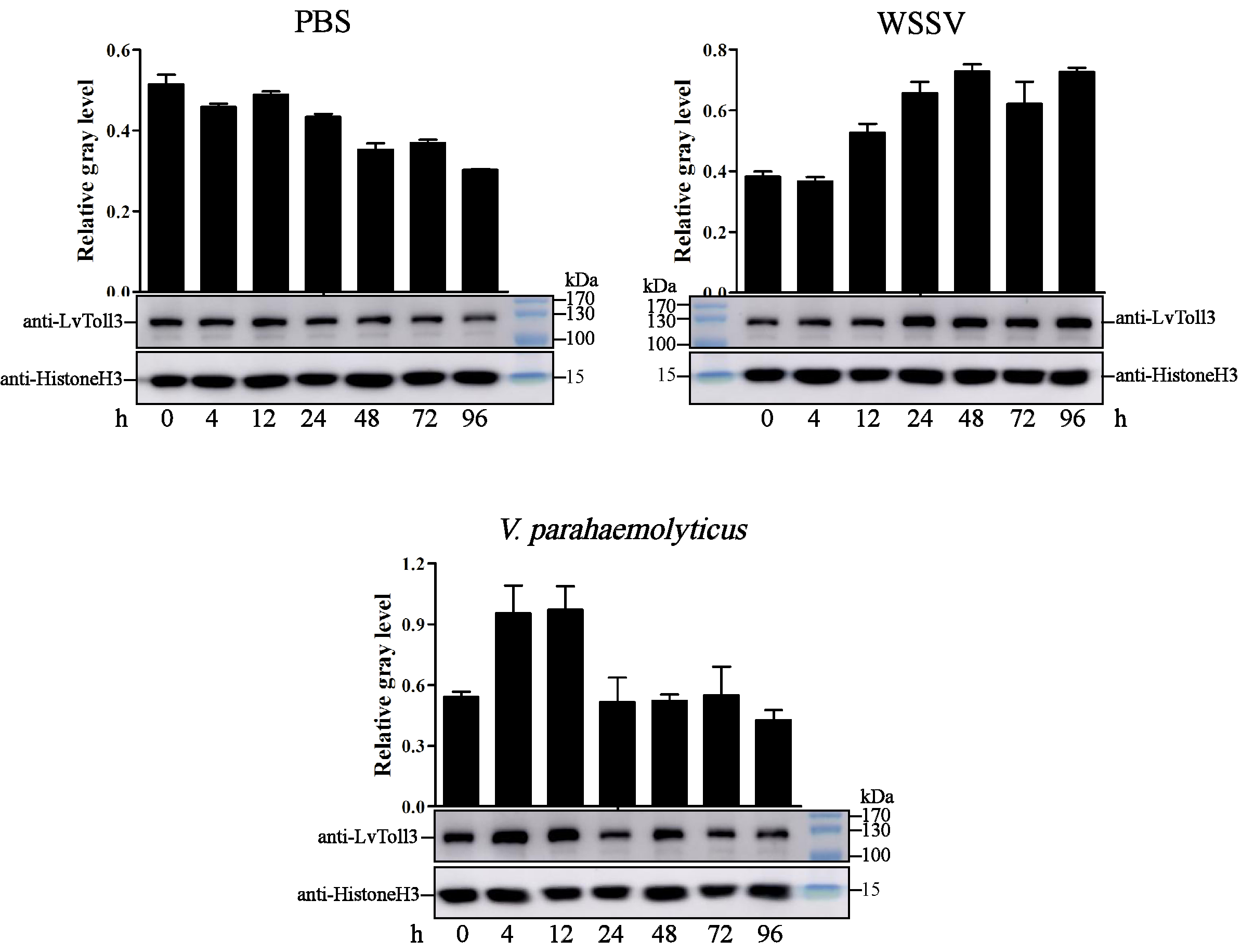

Supplement: Supplementary Figure 1 — Western blot analysis of the protein level of LvToll3 after PBS, WSSV, and V. parahaemolticus injections, respectively. The protein levels of LvToll3 were normalized to those of the internal control Histone H3. Each bar is mean ± SD of three independent quantification of the electrophoretic bands, **p < 0.01 and *p < 0.05 by two-tailed unpaired Student’s t-test. [file Image_1.tif]

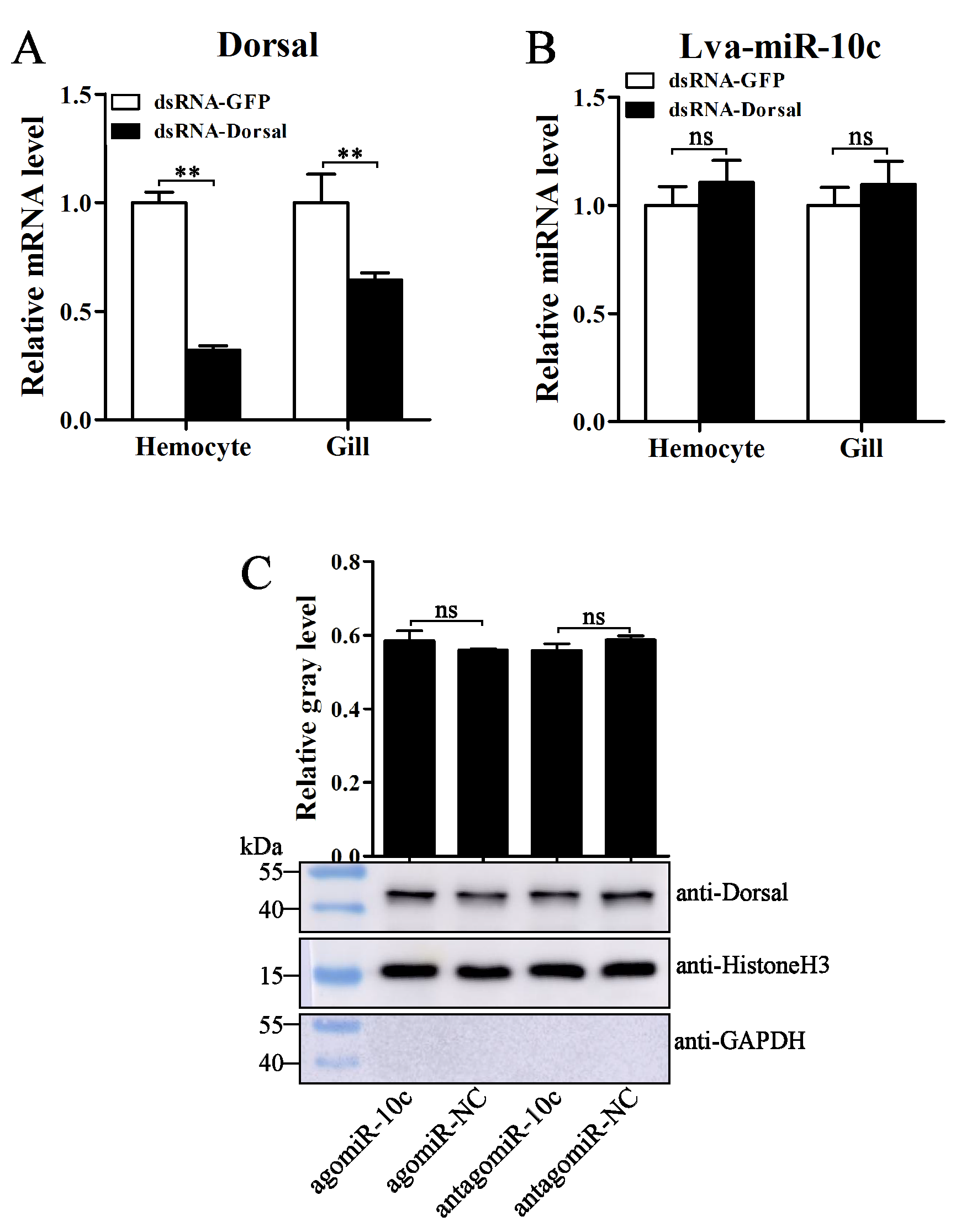

Supplement: Supplementary Figure 2 — Regulatory relationship between miR-10c and dorsal. (A) qRT-PCR analysis of the knockdown efficiency of dorsal. (B) Stem-loop qRT-PCR analysis of miR-10c expression in hemocyte and gill after the knockdown of dorsal. Values in the dsRNA-GFP control group were set as the baseline (1.0). Each bar represents the mean ± SD (n = 4), **p < 0.01 by two-tailed unpaired Student’s t-test. (C) Western blot analysis of dorsal expression in hemocyte and gill after treatment with miR-10c mimics and inhibitor in shrimp. The protein levels of dorsal protein were normalized to those of the internal control Histone H3. Each bar is mean ± SD of three independent quantifications of the bands, **p < 0.01 and *p < 0.05 by two-tailed unpaired Student’s t-test. [file Image_2.tif]
